# Synthesis, Structure, and Characterization of Thiacalix[4]-2,8-thianthrene

**DOI:** 10.3390/molecules28145462

**Published:** 2023-07-17

**Authors:** Masafumi Ueda, Moe Isozaki, Yasuhiro Mazaki

**Affiliations:** Department of Chemistry, Graduate School of Science, Kitasato University, Sagamihara 252-0373, Kanagawa, Japan; isozaki.moe@st.kitasato-u.ac.jp

**Keywords:** macrocycles, molecular tiling, solvent recognition, thiacalixarenes, thianthrene, fluorescence/phosphorescence dye

## Abstract

Sulfur-containing macrocycles have attracted substantial interest because they exhibit unique characteristics due to their polygonal ring-shaped skeleton. In this study, a thianthrene-based cyclic tetramer with the sulfur linker, thiacalix[4]-2,8-thianthrene (TC[4]TT), was successfully prepared from a cyclo-*p*-phenylenesulfide derivative using acid-induced intramolecular condensation. Single crystal X-ray diffraction revealed that TC[4]TT adopts an alternative octagonal form recessed to the inner side. Its internal cavity included small solvents, such as chloroform and carbon disulfide. Due to its polygonal geometry, TC[4]TT laminated in a honeycomb-like pattern with a porous channel. Furthermore, TC[4]TT showed fluorescence and phosphorescence emission in a CH_2_Cl_2_ solution at ambient and liquid nitrogen temperatures. Both emission bands were slightly redshifted compared with those of the reference compounds (di(thanthren-2-yl)sulfane (TT_2_S) and thianthrene (TT)). This work describes a sulfur-containing thiacalixheterocycle-based macrocyclic system with intriguing supramolecular chemistry based on molecular tiling and photophysical properties in solution.

## 1. Introduction

Thiacalixarenes are categorized as thioether-type macrocyclic compounds, which can adopt unique polygonal geometries by connecting aromatic hydrocarbons with sulfur linkers [1,2]. The compounds possess specific internal cavities formed by polygonal frameworks, depending on the number of building blocks, and demonstrate unusual inclusiveness. Based on their structural features, thiacalixarenes have been extensively studied as a platform of host–guest chemistry, such as the extraction of metal ions [3,4,5,6,7,8,9,10], transport materials [11], catalysts [12], crystal engineering/supramolecular architectures [13,14,15,16,17,18,19,20,21,22,23,24,25,26,27,28,29,30], guest adsorption materials [31,32,33], and chemosensors [34,35,36,37,38,39,40,41,42,43,44,45,46,47]. Hence, thiacalixarenes show potential as highly useful molecular skeletons for porous molecular recognition materials [48]. Furthermore, a new breed of calixarene derivatives built with aromatic heterocycles, e.g., thiophene [49,50,51], dithienothiophene [52], pyridine [53,54,55,56], pyrimidine [57], and triazine [58], have been synthesized. These derivatives have attracted much attention due to their proper inclusiveness, electrochemical properties, and molecular geometries based on the introduction of heteroatoms. We recently reported the synthesis of a sulfur-double bridged acene-based thiacalixarene derivative and revealed that its crystal structure shows honeycomb-like packing due to its unique hexagonal geometry and solvent inclusion within its internal cavity [59]. Embedding the folded thiaacenes into thiacalixarenes provides further polygonality and rigidity. Due to molecular tiling in the solid-state, porous channels utilizing a macrocyclic scaffold should form. This molecular design will be necessary for the development of sulfur-rich organic functional solid materials. However, there have been few reports on large thiacalixarenes composed of bicyclic or higher heterocycles, and the insight is insufficient.

**Figure 1:**
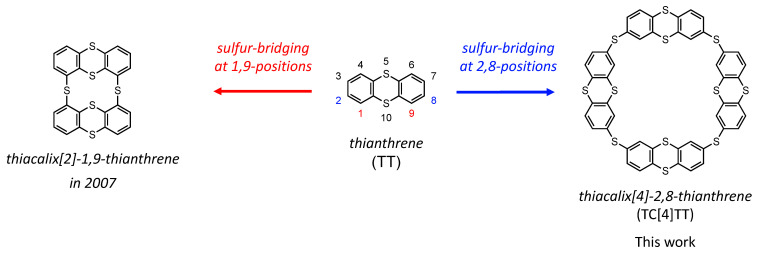
Molecular structures of thianthrene and thianthrene-based macrocyclic derivatives.

We focus on the 1,4-dithiin-embedded heterocycle compound, thianthrene (TT; Figure 1 (center)), as a building block. TT is a nonplanar heterocycle bent by sulfur. When developing sulfur-rich π-electron systems, it is important to attain remarkable electrochemical [60,61,62,63,64], photophysical [65,66,67], and molecular dynamics properties [68,69]. Parola et al. (2007) successfully synthesized the smallest family, thiacalix[2]-1,9-thianthrene (Figure 1 (left)), which was bridged at the 1,9-positions of the TT unit, from a thiacalix[4]arene derivative under high-temperature [70]. They revealed that this macrocycle adopts a flattened cone conformation with a concave internal cavity that can capture metal cations [71]. Conversely, investigating how the number of TT units and the ring sizes act on their characteristics is important for clarifying the features of this family. TT units should be connected at other sides instead of 1,9-positions to expand the macrocyclic ring sizes. Thus, we attempt to synthesize a larger cyclic tetramer bridged at the 2,8-positions of TT, thiacalix[4]-2,8-thianthrene (TC[4]TT; Figure 1 (right)).

While the macrocyclization of the smallest components is among the facile methods, for TC[4]TT synthesis, the preparation of 2,8-dihalogenated TT is not simple due to the formation of a regioisomer [72]. Conversely, some groups have certified that the acid-induced intramolecular condensation reaction [73] is advantageous for the formation of the TT skeleton in macrocyclic systems [59,74,75]. These results motivated us to induce TC[4]TT from the cyclo-*p*-phenylenesulfide derivative. Herein, we report the synthesis, crystal structure, and properties of TC[4]TT and its related compounds. TC[4]TT adopted an alternative concave octagonal structure and included relatively small solvents in the internal cavity. This macrocycle formed a honeycomb-type arrangement and channel stacking based on a polygonal structure with a hollow crystal structure. Furthermore, TC[4]TT demonstrated fluorescence and phosphorescence emission in the diluted solution as with thianthrene derivatives.

## 2. Results and Discussion

### 2.1. Synthesis

**Scheme 1:**
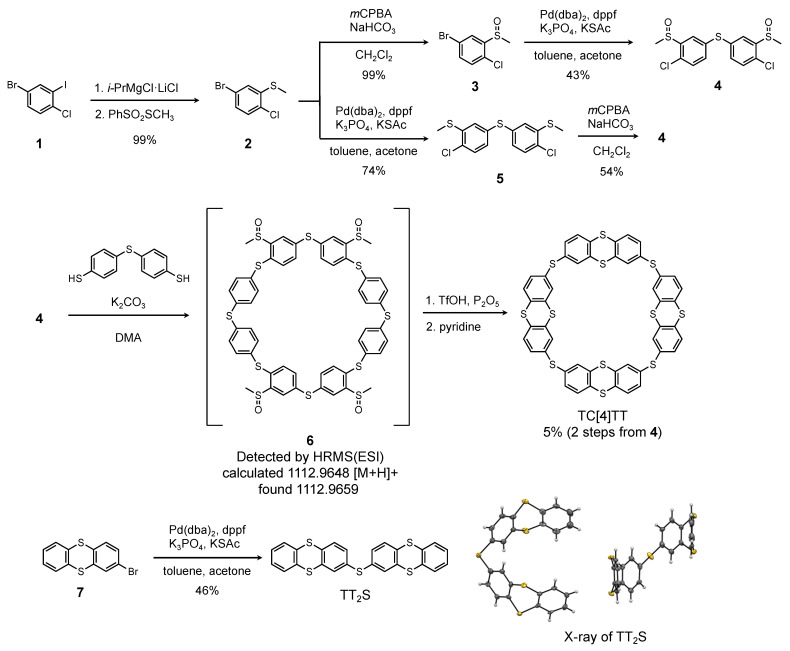
Synthesis of TC[4]TT and its reference compound TT_2_S. *m*CPBA: *m*-chloroperoxybenzoic acid; dba: dibenzylideneacetone; dppf: 1,1′-bis(diphenylphosphino)ferrocene; KSAc: potassium thioacetate; DMA: N,N’-dimethylacetamide; TfOH: trifluoromethanesulfonic acid.

The synthetic route used to generate TC[4]TT is illustrated in Scheme 1. 4-Bromo-1-chloro-2-iodobenzene (**1**) was treated with turbo Grignard reagent to selectively translate the iodine of **1**, and then, the generated arylmagnesium compound was reacted with *S*-methyl benzenesulfonothioate as an electrophile to afford (5-bromo-2-chlorophenyl)(methyl)sulfane (**2**) quantitatively. When Compound **2** was oxidized using *m-*chloroperbenzoic acid (*m*CPBA), 4-bromo-1-chloro-2-(methylsulfinyl)benzene (**3**) was obtained quantitatively. Subsequently, a Pd-catalyzed homocoupling reaction using potassium thioacetate as a sulfur source [76] of **3** afforded symmetrical diarylsulfide, bis(4-chloro-3-(methylsulfinyl)phenyl)sulfane (**4**), in 43% yield. Conversely, applying this Pd-mediated coupling reaction to **2** yielded bis(4-chloro-3-(methylthio)phenyl)sulfane (**5**) in a good yield. Although subsequent oxidation of **5** applying 1.5 equivalents of *m*CPBA generated **4** in 54% yield, a byproduct in which the sulfur linker was also oxidized was obtained in 14% yield. In both routes, the yield in two steps from Compound **2** was ca. 40%. In the next step, we conducted the macrocyclization of prepared **4** and 4,4′-thiobisbenzenethiol and detected the presence of cyclo-*p*-phenylenesulfide precursor **6** by electrospray ionization mass spectrometry (ESI-MS, Figure S13). However, separating and purifying this compound was difficult because the obtained crude products included polymer macromolecules. Hence, the crude product was used without further purification. Finally, the synthesis of TC[4]TT was achieved by intramolecular condensation using trifluoromethanesulfonic acid followed by demethylation of pyridine in 5% yield (from Compound **4**). High-resolution mass spectrometry (HRMS) detected its ion peak at *m*/*z =* 983.8527. The ^1^H nuclear magnetic resonance (NMR) spectrum provided simple peaks that could be associated with the AB-X style of the 2,8-substituted TT unit (Figure S11), and similarly, six carbon signals of the TT unit were observed in the ^13^C NMR spectrum (Figure S12). Thus, these spectroscopies revealed that TC[4]TT exhibits high-symmetric geometry due to a macrocyclic skeleton. Moreover, TC[4]TT showed considerably poor solubility in common organic solvents. Finally, the molecular structure of TC[4]TT was determined by single-crystal X-ray analysis, as will be described later. In this intramolecular reaction, other products which are presumed to be cyclic hexamer or larger oligomers, were obtained in trace amounts. Furthermore, the reference linear dimer, di(thianthrene-2-yl)sulfane (TT_2_S), was obtained from prepared 2-bromothianthrene (**7**) by Pd-mediated coupling in 46% yield. This linear dimer adopted a U-shaped geometry in the crystal structure.

### 2.2. Crystal Structure

**Figure 2:**
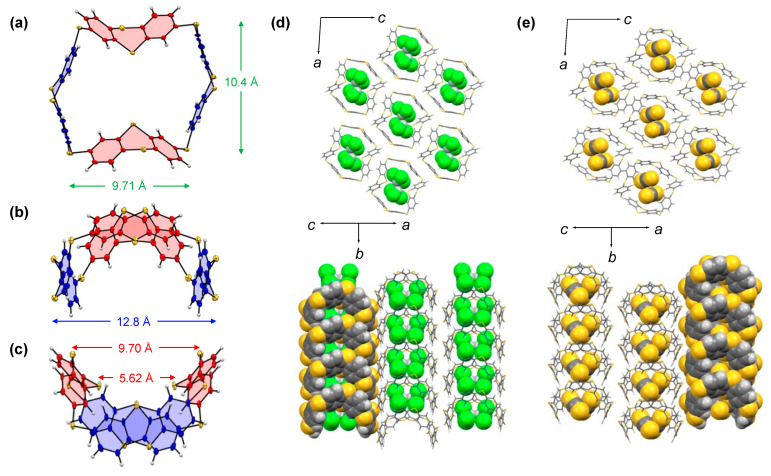
Molecular structure of TC[4]TT in (TC[4]TT)(CHCl_3_)_2_: (**a**) top view; (**b**) side view I; and (**c**) side view II. Ellipsoids represent a 50% probability. Crystal packing diagrams of (**d**) (TC[4]TT)(CHCl_3_)_2_ and (**e**) (TC[4]TT)(CS_2_)_2_.

Single crystals of TC[4]TT were obtained by slowly evaporating CHCl_3_ and CS_2_. Both crystals belonged to the *I*2 space group with a monoclinic crystal system. The inwardly folded (Figure 2a, painted in red) and protruding TT (painted in blue) units alternatively shaped a macrocyclic skeleton and adopted concave octagonal geometry. Interestingly, this conformer was energetically unstable as a single molecule in vacuo compared with its structural isomer in which all TT units are flared, and the estimated total energy difference was ca. 0.13 kcal mol^−1^ (Figure S22). The folding angles of the constituent TT units were 124.9(1) and 136.1(1)°. The inwardly folded TT bent sharply compared with the linear dimer TT_2_S (corresponding angles: 137.1(1) and 130.4(1)°). The TT units distorted as a result of macrocyclization. The distances between the sulfur bridges were 9.71 and 10.4 Å, respectively. Although the distance between two flared TT units was 12.8 Å, the distance was 5.62 Å at the narrowest part and 9.70 Å at the widest parts of the collapsed TT units (Figure 2b,c). Thus, TC[4]TT possesses a specially shaped internal cavity. In the crystal structure, the solvents (CHCl_3_ and CS_2_) were included in the cavity of TC[4]TT, and the composition ratio of compound and solvent was 1:2 (Figure 2d,e; (TC[4]TT)(CHCl_3_)_2_ and (TC[4]TT)(CS_2_)_2_). For CHCl_3_ and CS_2_, the two molecules in the cavity fit in a V-shape along with the lamination direction. Although we attempted to incorporate solvents with different sizes, such as benzene, 1,4-dioxane, and chlorobenzene, into TC[4]TT, parsable crystals were not obtained. In donor–acceptor-type complex formations with C_60_ and C_70_, distinct complexation could not be observed. This result shows that TC[4]TT can selectively capture relatively small molecules in the crystal state. Thus, TC[4]TT can expect selective inclusion with small organic molecules and metal ions similar to thiacalixarene derivatives. Notably, intermolecular interactions between TC[4]TT and each encapsulated solvent were not observed, suggesting that the solvent molecules just fit in the cavity. Conversely, differences were observed in the intermolecular packing forces acting among TC[4]TT, while (TC[4]TT)(CHCl_3_)_2_ and (TC[4]TT)(CS_2_)_2_ were the same crystal system. The clathrate crystal of TC[4]TT and CHCl_3_ contains some C–H···π interactions and C–H···S contacts [77] between four adjacent molecules. Its columnar stacking was also built by C–H···π interactions along the *ac* axis and C–H···S contacts along the *b* axis (Figure S15). In comparison, (TC[4]TT)(CS_2_)_2_ was formed by some atomic contacts, such as C–C, C–S, and S–S contacts, in addition to these interactions (Figure S17). The density of the crystals was 1.591 for (TC[4]TT)(CHCl_3_)_2_ and 1.491 for (TC[4]TT)(CS_2_)_2_. The crystal of (TC[4]TT)(CS_2_)_2_ was packed more densely than that of (TC[4]TT)(CHCl_3_)_2_. Thus, coupled with the pseudo hexagonal geometry consisting of imminent sulfur of TT and the linker, TC[4]TT was arranged in a honeycomb style with a channel structure due to molecular tiling. These results suggest that TC[4]TT exhibits adsorption and transport properties and shows potential as an organic porous solid material.

### 2.3. Photophysical Properties

**Figure 3:**
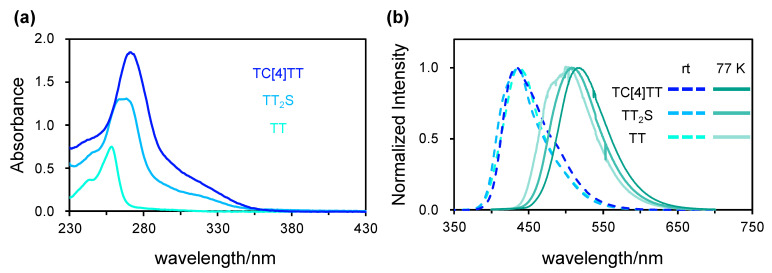
(**a**) Absorption spectra of TC[4]TT, TT_2_S, and TT in a CH_2_Cl_2_ solution. (**b**) Emission spectra of TC[4]TT, TT_2_S, and TT in a CH_2_Cl_2_ solution at room temperature and 77 K. The solution concentration is 2.0 × 10^−5^ mol L^−1^. The excitation wavelengths are 271 (TC[4]TT), 268 (TT_2_S), and 258 nm (TT). Phosphorescence spectra at 77 K were measured with a delay of 15 ms.

**Figure 4:**
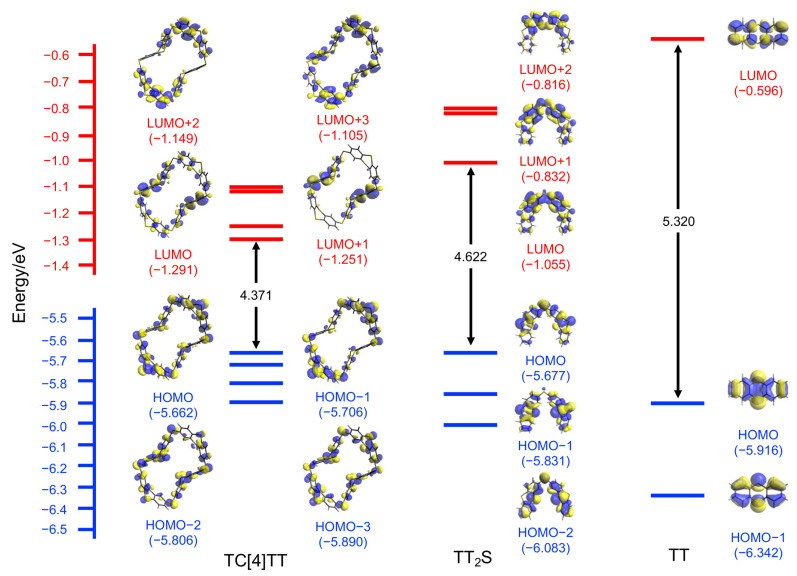
Calculated molecular orbitals of TC[4]TT, TT_2_S, and TT at the RB3LYP/6-31G(d,p) level.

We investigated the photophysical properties of TC[4]TT and TT_2_S because these compounds showed fluorescence emission on thin-layer chromatography (TLC) plates during purification. The CH_2_Cl_2_ solution of TC[4]TT (*c* = 2.0 × 10^−5^ mol L^−1^) showed an absorption maximum at 271 nm with broad shoulder bands at 290–360 nm, which could be ascribed to the π–π* transitions (Figure 3a). The molar extinction coefficient (*ε*) at λ_max_ = 271 nm was 92,500 L mol^−1^ cm^−1^, higher than those of TT_2_S and TT (65,500 and 37,600 L mol^−1^ cm^−1^, respectively), depending on the number of TT units. Furthermore, the absorption wavelength of TC[4]TT was slightly redshifted compared with those of TT_2_S and TT (λ_max_ = 268 and 258 nm). This redshift stems from a decline in the gap between the highest occupied molecular orbital (HOMO) and the lowest unoccupied molecular orbital (LUMO), as shown in Figure 4. The simulated absorption spectrum of TC[4]TT by the quantum chemical calculation also agreed with the experimental result (time-dependent density-functional theory calculation at the RB3LYP/6-31(d,p) level, Figure S20). The absorption bands were composed primarily of S_0_ → S_2_, S_3_, and S_8_ electronic transitions (Table S4). This result suggested that widely and circularly distributed HOMOs and LUMOs based on the macrocyclic skeleton, including the sulfur linkers, could affect the electronic transitions. In addition, the quantum chemical calculation supported that the energy gaps of S_0_ → S*_n_* (*n* > 1) of TC[4]TT were lower than those of TT_2_S (Figure S21 and Table S5).

TC[4]TT generates a blue emission on the TLC plate under irradiation with a UV lamp (λ = 365 nm). In a dilute CH_2_Cl_2_ solution, emission bands at λ_max_ = 447 nm were observed under ambient conditions (Figure 3b). Similarly, TT_2_S exhibited emission bands at λ_max_ = 446 nm. These compounds showed insignificantly redshifted emission bands compared to that of TT (λ_max_ = 436 nm). The quantum yields (Φ) of TC[4]TT and TT_2_S were 7 and 6%, respectively. Although these values were slightly higher than that of TT (Φ = 3%), the emission efficiency remained low. Thus, the nonradiative process mostly results from the geometric vibrations of the TT unit at the excitation despite the advantages of a rigid skeleton generated from macrocyclization. That is, TC[4]TT possesses a highly flexible molecular geometry.

Subsequently, a frozen CH_2_Cl_2_ solution of TC[4]TT at liquid nitrogen temperature (77 K) showed a new emission band at λ_max_ = 516 nm in the long wavelength region. The reference compounds TT_2_S and TT also exhibited redshifted emission bands at λ_max_ = 508 and 504 nm at 77 K. These low-temperature spectra were measured with a 15 ms delay, and the lifetimes were 45 ms for TC[4]TT and 34 ms for TT_2_S, which could be identified as phosphorescence emission. The phosphorescence emission maximum of TC[4]TT was gradually redshifted, which could indicate that the energy gap of the lowest singlet (S_1_) and triplet excited state (T_1_) was narrower than those of TT_2_S and TT. The efficient intersystem crossing process might be prompted by rigidity arising from macrocyclization with the sulfur linker. The quantum chemical calculation supported that the energy gap between S_1_ and T_1_ of TC[4]TT is 0.629 eV. This was lower than those of TT_2_S (0.795 eV) and TT (0.751 eV, Figure S23 and Table S6). We found that TC[4]TT shows potential as an organic phosphorescence characteristic, as with other thianthrene derivatives. Macrocylization of chromophores might be a useful molecular design for pure organic phosphorescence materials. Room-temperature phosphorescence behavior on crystals based on molecular tiling is currently under investigation.

## 3. Conclusions

In conclusion, we successfully prepared a thianthrene-based cyclic tetramer linked with sulfur, thiacalix[4]-2,8-thianthrene (TC[4]TT), by acid-induced intramolecular condensation of a cyclo-*p*-phenylenesulfide derivative. In addition, we clarified its molecular structure and its fundamental photochemical properties. TC[4]TT adopts a cage-shaped and concave octagonal geometry due to four bent thianthrenes and four sulfur linkers in the crystal state. Furthermore, based on its polygonal skeleton with an internal cavity, this compound forms a unique honeycomb-style channel architecture stemming from molecular tiling. Small solvent molecules (CHCl_3_ and CS_2_) are included in the cavity, which exhibits unusual inclusiveness, as with thiacalixarene derivatives. That is, the compound can be applied to adsorbed porous materials. Intriguingly, the diluted CH_2_Cl_2_ solution of TC[4]TT demonstrates fluorescence emission at ambient temperature and phosphorescence emission at liquid nitrogen temperature. Notably, phosphorescence emission bands are redshifted compared to those of thianthrene and its sulfur-bridged dimer. This result indicates that macrocyclization by a sulfur bridge might load an efficient intersystem crossing system for phosphorescence emission. Thus, we found that TC[4]TT can be utilized as a chemical sensor. Molecular recognition ability, solid-state phosphorescence emission, and electrochemical properties are currently being studied.

## 4. Materials and Methods

### General Information

All reagents were commercially sourced. Melting points were determined using a Yanaco MP-500P micro melting point apparatus. ^1^H (400 or 600 MHz) and ^13^C (100 or 150 MHz) nuclear magnetic resonance spectra were recorded using Bruker AVANCE 400 and 600 instruments. The following abbreviations were used to describe the multiplicities: singlet (s); doublet (d); doublet of doublets (dd); and multiplet (m). The diffraction data for TT_2_S (100.15 K, ccdc#: 2270347), (TC[4]TT)(CHCl_3_)_2_ (100.15 K, ccdc#: 2270345), and (TC[4]TT)_2_(CS_2_)_2_ (100.15 K, ccdc#: 2270346) were collected using a Rigaku XtaLAB Synergy-S system with multilayer mirror-monochromatized Cu*K*a radiation (l = 1.54184 Å) at Kitasato University. Raw frame data were integrated using CrysAlisPro software [78]. Using Olex2 [79], the crystal structures were solved employing SHELXT [80] and refined using full-matrix least squares in *F*^2^ (SHELXL) [81]. Absorption spectra were recorded using a JASCO (JASCO corporation, Tokyo, Japan) V-560 instrument. Fluorescence spectra were collected using a JASCO FP-8550 spectrofluorometer. Fluorescence quantum yields (Φ) were confirmed using a JASCO ILF-135 | 120 mm dia. *Φ* Integrating Sphere. Phosphorescence spectra and lifetimes were collected using a JASCO FP-8550 spectrofluorometer combined with a PMU-130 liquid nitrogen cooling unit. IR spectra were recorded using a JASCO FT/IR-4600 spectrometer. High-solution mass spectrometry (HRMS) spectra were recorded on a Thermo Scientific Exactive Plus Orbitrap Mass spectrometer for ionization. Only relatively intense peaks were reported. All calculations were performed at the Research Center for Computational Science (Okazaki, Japan) using the Gaussian 16 Program (Revision C.01) [82]. The optimized structures were estimated at the RB3LYP/6-31G(d,p) level of theory. Frequency calculations were conducted to ensure that these structures were local minima. TD-DFT calculations based on the respective optimized structures were conducted using the RB3LYP/6-31G(d,p) level.

Synthesis of **2**. In a 200 mL two-necked recovery flask, 4-bromo-1-chloro-2-iodobenzene (**1**, 8.16 g, 25.7 mmol) and dry THF (30 mL) were mixed under argon atmosphere, and the mixture was cooled to −78 °C. The THF solution of *i*-PrMgCl·LiCl (1.3 M, 23.7 mL, 30.9 mmol) was slowly added and stirred for 12 h. The prepared magnesium reagent was added to a solution of prepared *S*-methylbenzenesulfonothioate (5.33 g, 28.3 mmol) [83] in dry THF (15 mL) at −78 °C. The resulting solution was stirred at ambient temperature for 2 h and then quenched with sat. NH_4_Claq., extracted with CH_2_Cl_2_ and dried over anhydrous Na_2_SO_4_. The organic solution was concentrated under reduced pressure. The residue was purified by column chromatography on silica gel using hexane as an eluent to afford (5-bromo-2-chlorophenyl)(methyl)sulfane (**2**, 6.04 g, 25.4 mmol) as a colorless block in 99% yield. Mp = 54–55 °C. ^1^H NMR (600 MHz, CDCl_3_) δ 7.22–7.21 (m, 1H), 7.18 (d, *J* = 1.2 Hz, 2H), 2.48 (s, 3H). ^13^C NMR (150 MHz, CDCl_3_) δ 140.4, 130.60, 130.56, 128.4, 127.8, 121.1, and 15.3. UV–vis (CH_2_Cl_2_, *c* = 1.0 × 10^−5^ M) λ_max_ (*ε*) 258 (8000) nm. IR (KBr) 3027, 2991, 2921, 2369, 1866, 1677, 1569, 1543, 1446, 1369, 1318, 1249, 1139, 1123, 1110, 1092, 1076, 1030, 956, 841, 795, 778, 671, 539, and 426 cm^−1^. HRMS (ESI, positive mode): *m*/*z* calcd for C_7_H_6_ClBrS [M]^+^ 235.9057; found 235.9058.

Synthesis of **3**. Compound **2** (1.00 g, 4.21 mmol), *m*CPBA (1.12 g, 4.21 mmol), NaHCO_3_ (357 mg, 4.21 mmol), and CH_2_Cl_2_ (50 mL) were mixed in a 200 mL recovery flask. The mixture was stirred at 0 °C for 2 h. The reaction mixture was quenched with Na_2_S_2_O_3_aq., and then the organic layer was extracted with CH_2_Cl_2_ and dried over MgSO_4_. After filtration, the solution was evaporated under reduced pressure. The residue was purified by column chromatography on silica gel using EtOAc as an eluent to afford **3** (1.06 g, 4.21 mmol) as a colorless block in 99% yield. Mp = 77–78 °C. ^1^H NMR (400 MHz, CDCl_3_) δ 8.07 (d, *J* = 1.6 Hz, 1H), 7.57 (dd, *J* = 1.6 and 8.4 Hz, 1H), 7.27 (d, *J* = 8.4 Hz, 1H), 2.84 (s, 3H). ^13^C NMR (100 MHz, CDCl_3_) δ 145.6, 135.1, 131.2, 128.6, 128.4, 122.4, and 41.6. UV–vis (CH_2_Cl_2_, *c* = 2.0 × 10^−5^ M) λ_max_ (*ε*) 236 (12,000) nm. IR (KBr) 3072, 3053, 3006, 1697, 1443, 1430, 1366, 1302, 1233, 1142, 1104, 1072, 1058, 1023, 971. 955, 895, 812, 693, 681, 555, 542, 451, and 403 cm^−1^. HRMS (ESI, positive mode): *m*/*z* calcd for C_7_H_6_BrClOS [M + H]^+^ 252.9084; found 252.9087.

Synthesis of **4** from **3**. Compound **3** (537 mg, 2.12 mmol), Pd(dba)_2_ (61 mg, 0.11 mmol), dppf (82 mg, 0.15 mmol), KSAc (121 mg, 1.06 mmol), and K_3_PO_4_ (270 mg, 1.27 mmol) were added to a 30 mL Schlenk tube in toluene (1.1 mL) and acetone (0.5 mL) under an argon atmosphere. The reaction mixture was stirred for 6 h under reflux. After cooling to ambient temperature, the suspension was quenched with NH_4_Claq. The aqueous layer was extracted with Et_2_O, and the organic layer was washed with brine and dried over MgSO_4_. The organic phase was passed through celite and concentrated. The residue was purified by column chromatography on silica gel using EtOAc as an eluent to afford **4** (174 mg, 0.46 mmol) as a colorless needle in 43% yield. Mp = 145–147 °C. ^1^H NMR (400 MHz, CDCl_3_) δ 7.93 (d, *J* = 2.0 Hz, 1H), 7.88 (d, *J* = 2.0 Hz, 1H), 7.43–7.34 (m, 4H), 2.84 (s, 3H), 2.83 (s, 3H). ^13^C NMR (100 MHz, CDCl_3_) δ 145.3, 145.2, 136.14, 136.13, 134.3, 134.2, 130.94, 130.88, 129.13 129.10, 127.5, 127.4, 41.8, and 41.7. UV–vis (CH_2_Cl_2_, *c* = 2.0 × 10^−5^ M) λ_max_ (*ε*) 290 (10,500), 259 (21,400) nm. IR (KBr) 3446, 3065, 2996, 2912, 1636, 1567, 1445, 1415, 1365, 1291, 1247, 1146, 1088, 1062, 1023, 960, 897, 830, 699, 557, 459, 413, and 402 cm^−1^. HRMS (ESI, positive mode): *m*/*z* calcd for C_14_H_12_Cl_2_O_2_S_3_ [M + H]^+^ 378.9449; found 378.9450.

Synthesis of **5**. Compound **2** (3.84 g, 16.2 mmol), Pd(dba)_2_ (465 mg, 0.81 mmol), dppf (628 mg, 1.13 mmol), KSAc (924 mg, 8.09 mmol), and K_3_PO_4_ (2.06 g, 9.70 mmol) were added to a 50 mL Schlenk tube in toluene (8.1 mL) and acetone (4.0 mL) under an argon atmosphere. The reaction mixture was stirred for 6 h under reflux. After cooling to ambient temperature, the suspension was quenched with NH_4_Claq. The aqueous layer was extracted with Et_2_O, and the organic layer was washed with brine and dried over MgSO_4_. The organic phase was passed through celite and concentrated. The residue was purified by column chromatography on silica gel using EtOAc/hexane (1:9, *v*/*v*) as the eluent to afford **5** (2.09 g, 6.02 mmol) as a colorless needle in 74% yield. Mp = 100–102 °C. ^1^H NMR (400 MHz, CDCl_3_) δ 7.26 (d, *J* = 8.0 Hz, 2H), 7.01 (d, *J* = 2.0 Hz, 2H), 6.99 (dd, *J* = 2.0 and 8.4 Hz, 2H), 2.41 (s, 6H). ^13^C NMR (100 MHz, CDCl_3_) δ 139.3, 134.5, 130.9, 130.0, 127.8, 127.3, and 15.1. UV–vis (CH_2_Cl_2_, *c* = 2.0 × 10^−5^ M) λ_max_ (*ε*) 260 (37,500) nm. IR (KBr) 3059, 2985, 2916, 1884, 1559, 1454, 1428, 1361, 1269, 1247, 1155, 1118, 1095, 1030, 956, 857, 845, 815, 674, 555, 464, and 434 cm^−1^. HRMS (ESI, positive mode): *m*/*z* calcd for C_14_H_12_Cl_2_S_3_ [M]^+^ 345.9473; found 345.9471.

Synthesis of **4** from **5**. Compound **5** (189 mg, 0.54 mmol), *m*CPBA (217 mg, 0.82 mmol), NaHCO_3_ (69 mg, 0.82 mmol), and CH_2_Cl_2_ (5 mL) were added to a 50 mL recovery flask. The mixture was stirred at 0 °C for 2 h. The reaction mixture was quenched with Na_2_S_2_O_3_aq., and then the organic layer was extracted with CH_2_Cl_2_ and dried over MgSO_4_. After filtration, the solution was evaporated under reduced pressure. The residue was purified by column chromatography on silica gel using EtOAc as an eluent to afford **4** (112 mg, 0.30 mmol) as a colorless block in 54% yield.

Compound **7** was prepared according to the bromination of thianthrene [84].

Synthesis of di(thianthren-2-yl)sulfane (TT_2_S). Compound **7** (433 mg, 1.47 mmol), Pd(dba)_2_ (42 mg, 0.07 mmol), dppf (57 mg, 0.10 mmol), KSAc (84 mg, 0.73 mmol), and K_3_PO_4_ (187 mg, 0.88 mmol) were added to a 30 mL Schlenk tube in toluene (2.0 mL) and acetone (1.0 mL) under an argon atmosphere. The reaction mixture was stirred for 12 h under reflux. After cooling to ambient temperature, the suspension was quenched with NH_4_Claq. The aqueous layer was extracted with CH_2_Cl_2_, and the organic layer was washed with brine and dried over MgSO_4_. The organic phase was passed through celite and concentrated. The residue was purified by column chromatography on silica gel using CH_2_Cl_2_/hexane (1:1, *v*/*v*) as the eluent to afford TT_2_S (155 mg, 0.34 mmol) as a colorless block in 46% yield. Mp = 149–150 °C. ^1^H NMR (400 MHz, CD_2_Cl_2_) δ 7.51–7.45 (m, 4H), 7.44 (d, *J* = 1.6 Hz, 2H), 7.41 (d, *J* = 8.0 Hz, 2H), 7.30–7.24 (m, 4H), 7.20 (dd, *J* = 2.0 and 8.0 Hz, 2H), ^13^C NMR (100 MHz, CDCl_3_) δ 136.9, 135.6, 135.3, 135.1, 135.0, 130.8, 130.3, 129.2, 128.9, 128.7, 127.9, and 127.7. UV–vis (CH_2_Cl_2_, *c* = 2.0 × 10^−5^ M) λ_max_ (*ε*) 268 (65,500) nm. IR (KBr) 3047, 1562, 1441, 1359, 1254, 1105, 876, 816, 743, 661, 592, 475, and 450 cm^−1^. HRMS (ESI, positive mode): *m*/*z* calcd for C_24_H_14_S_5_ [M]^+^ 461.9694; found 461.9691.

Synthesis of thiacalix[4]thianthrene (TC[4]TT). Compound **4** (303 mg, 0.80 mmol), 4,4′-thiobisbenzenethiol (200 mg, 0.80 mmol), K_2_CO_3_ (221 mg, 1.60 mmol), and dry DMA (64 mL) were added to a 100 mL Schlenk tube under an argon atmosphere. The mixture was stirred at 150 °C for 2 days. After cooling to ambient temperature, the mixture was poured into 10% acetic acid (160 mL). The water layer was extracted with CH_2_Cl_2_, and the organic phase was washed with NaHCO_3_aq. and then dried over MgSO_4_. The organic solution was evaporated under reduced pressure. After the ocherous powder was collected, 329 mg of crude product involving Compound **6** was obtained. In the next step, the crude product, P_2_O_5_ (77 mg, 0.54 mmol), and trifluoromethanesulfonic acid (3.5 mL) were added to a 50 mL two-necked recovery flask under an argon atmosphere. The reaction mixture was stirred at 65 °C for 2 days and then poured into ice water. After the precipitates were collected by filtration, the residue was dissolved in pyridine (25 mL) and refluxed for 24 h. The reaction mixture was cooled to ambient temperature, and MeOH was added. The resulting precipitate was collected and then subjected to column chromatography on silica gel using CS_2_ as the eluent to afford thiacalix[4]thianthrene (14 mg, 0.01 mmol) as a colorless block in 5% yield from Compound **4** in 2 steps. Mp = 203 °C (decomp.). ^1^H NMR (600 MHz, CS_2_/CDCl_3_) δ 7.31 (d, *J* = 7.8 Hz, 8H), 7.19–7.15 (m, 16H), ^13^C NMR (150 MHz, CS_2_/CDCl_3_) δ 136.6, 135.2, 134.5, 130.5, 130.0, and 128.9. UV–vis (CH_2_Cl_2_, *c* = 2.0 × 10^−5^ M) λ_max_ (*ε*) 271 (92,500) nm. IR (KBr) 1557, 1442, 1363, 1252, 1107, 905, 712, 731, and 450 cm^−1^. HRMS (ESI, positive mode): *m*/*z* calcd for C_48_H_24_S_12_ [M]^+^ 983.8521; found 983.8527.

## Data Availability

Not applicable.

## Supplementary Materials

The following supporting information can be downloaded at: https://www.mdpi.com/article/10.3390/molecules28145462/s1: Figures S1–S12: The copies of ^1^H and ^13^C NMR charts; Figure S13: ESIMS spectra of crude products in macrocyclization; Figures S14–S19: Molecular and crystal structures of (TC[4]TT)(CHCl_3_)_2_, (TC[4]TT)(CS_2_)_2_, and TT_2_S; Figures S20–S21: Simulated absorption spectra of TC[4]TT and TT_2_S; Figure S22: Optimized structures of TC[4]TT and its structural isomer; Figure S23: TD-DFT calculated energy diagrams at singlet (S_1_) and triplets (T_n_) for TC[4]TT, TT_2_S, and TT; Table S1: Crystal data for (TC[4]TT)(CHCl_3_)_2_, (TC[4]TT)(CS_2_)_2_, and TT_2_S; Tables S2–S3: Selected bond lengths of TT units of (TC[4]TT)(CHCl_3_)_2_ and (TC[4]TT)(CS_2_)_2_; Tables S4–S5: Calculated photophysical data for TC[4]TT and TT_2_S; Table S6: TD-DFT calculated energy levels at S_1_ and T_n_ for TC[4]TT, TT_2_S, and TT. References [85,86,87] are cited in the supplementary materials.

## Sample Availability

Samples of the compounds are available from the authors.
