# Supplementary material for: Synthesis, Structure, and Characterization of Thiacalix[4]-2,8-thianthrene
Source: Molecules. 2023 Jul 17;28(14):5462. doi: 10.3390/molecules28145462 (PMC10383442; doi:10.3390/molecules28145462)
Supplement: Supplementary file 1 [file molecules-28-05462-s001.zip › molecules-2500322-supplementary.pdf]

Supporting Information

Synthesis, Structure, and Characterization of Thiacalix[4]-2,8-Thianthrene

Masafumi Ueda*, Moe Isozaki, Yasuhiro Mazaki*

Department of Chemistry, Graduate School of Science, Kitasato University, 1-15-1 Kitazato, Minami-ku, Sagamihara, Kanagawa 252-0373, Japan

Contents

1. **^{1}H and ^{13}C NMR Charts** **1**
2. **ESIMS Spectra** **7**
3. **Crystal Data for TC[4]TT and TT_2S** **8**
4. **Photophysical Data** **12**
5. **Theoretical Study** **14**
6. **References** **16**

1. ^{1}H and ^{13}C NMR Charts

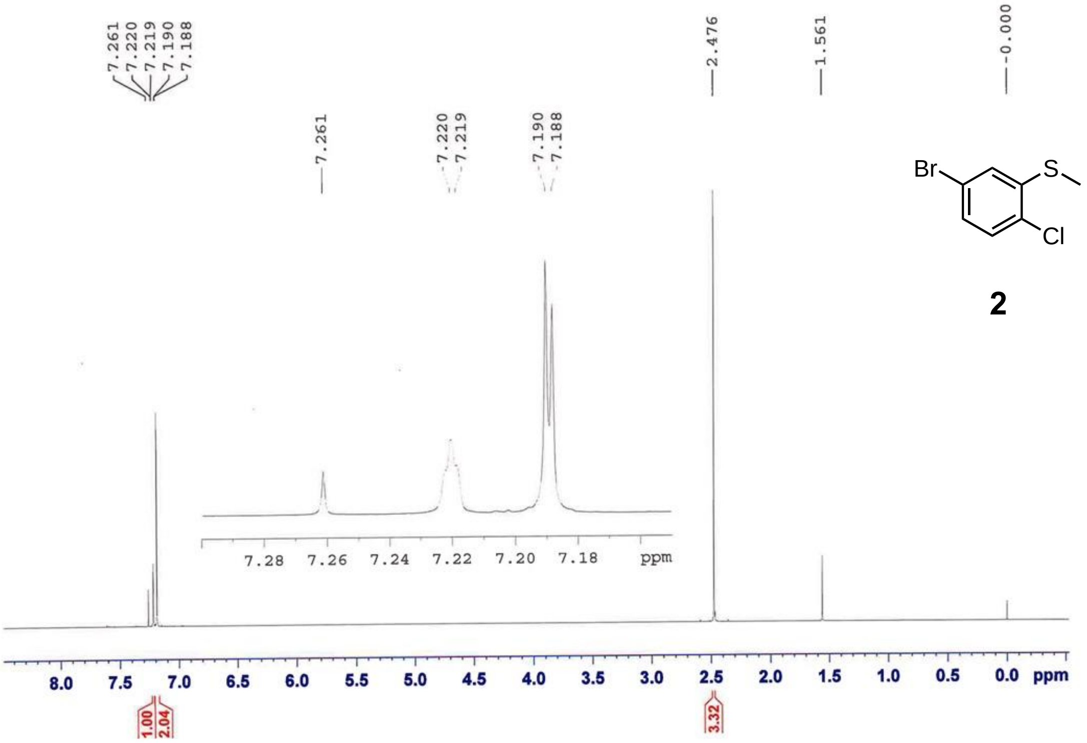

Figure S1. ^{1}H NMR spectrum of **2** (600 MHz, $CDCl_3$).

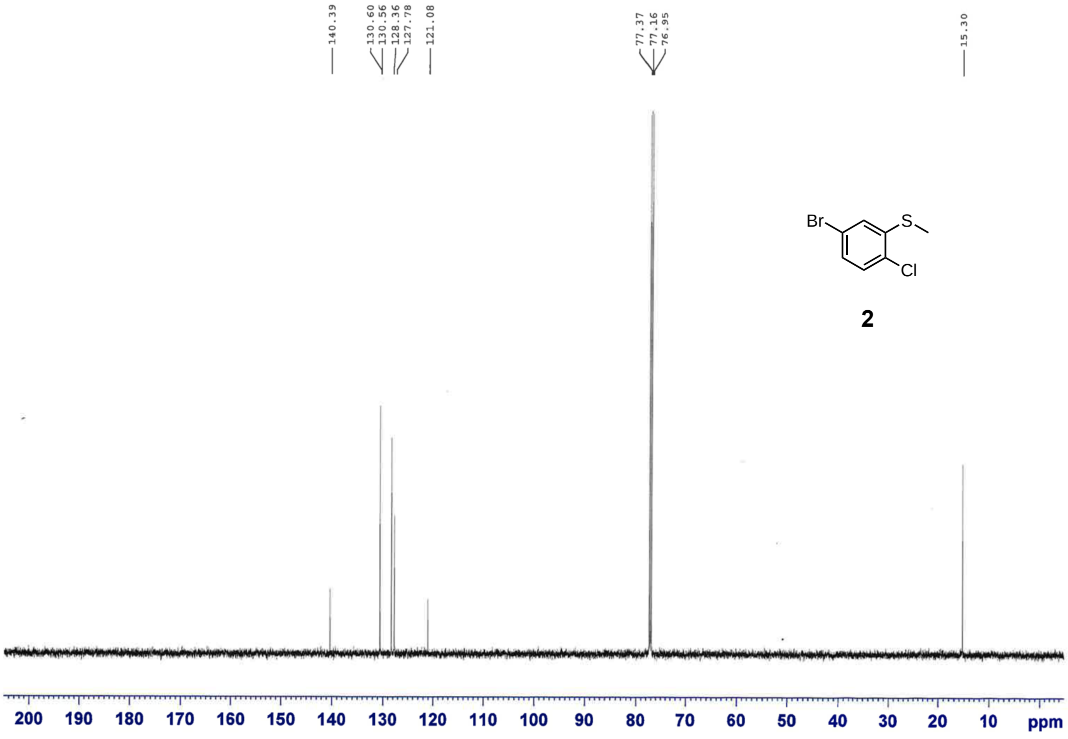

Figure S2. ^{13}C NMR spectrum of **2** (150 MHz, $CDCl_3$).

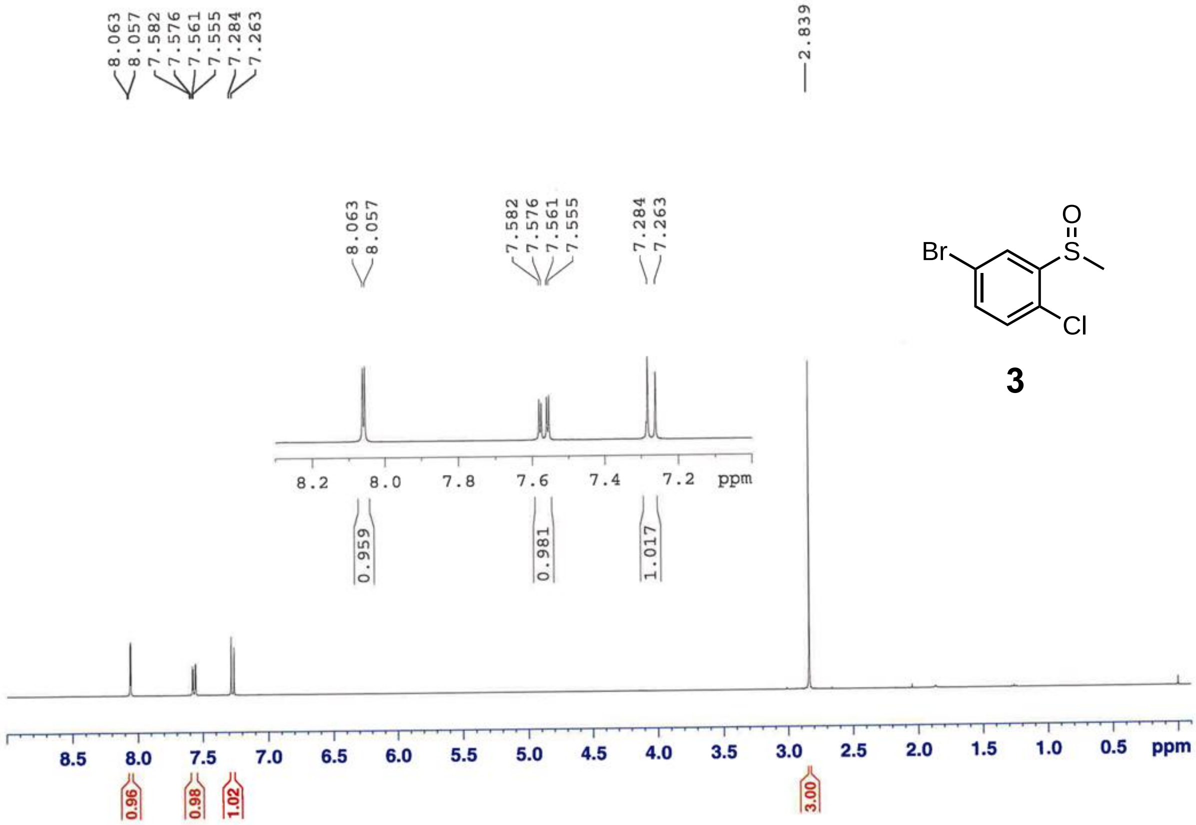

Figure S3. ^{1}H NMR spectrum of **3** (400 MHz, $CDCl_3$).

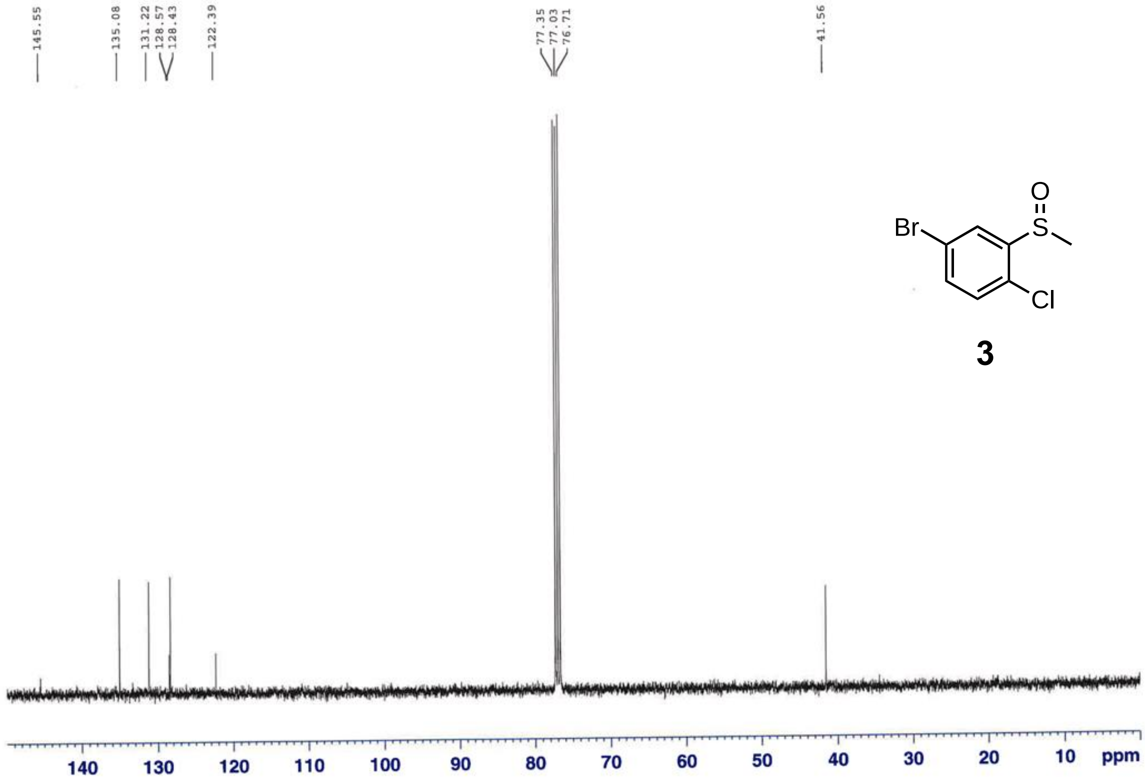

Figure S4. ^{13}C NMR spectrum of **3** (100 MHz, $CDCl_3$).

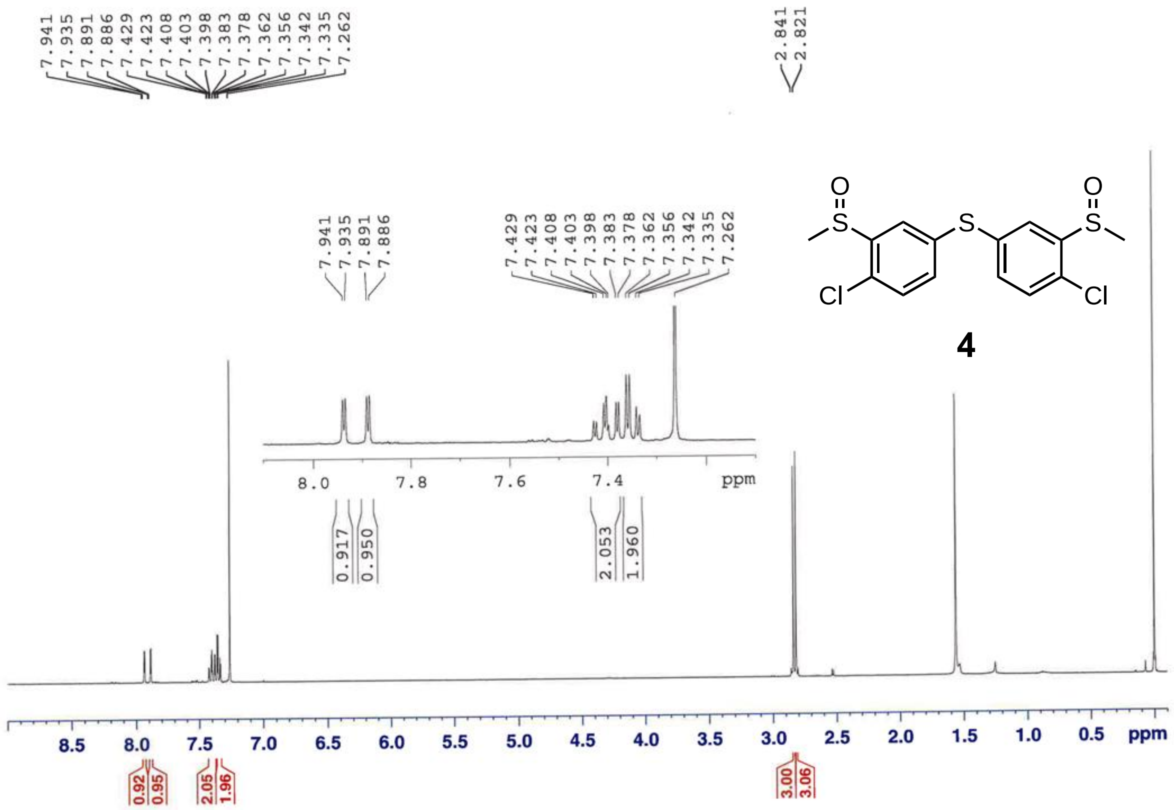

Figure S5. ^{1}H NMR spectrum of **4** (400 MHz, $CDCl_3$).

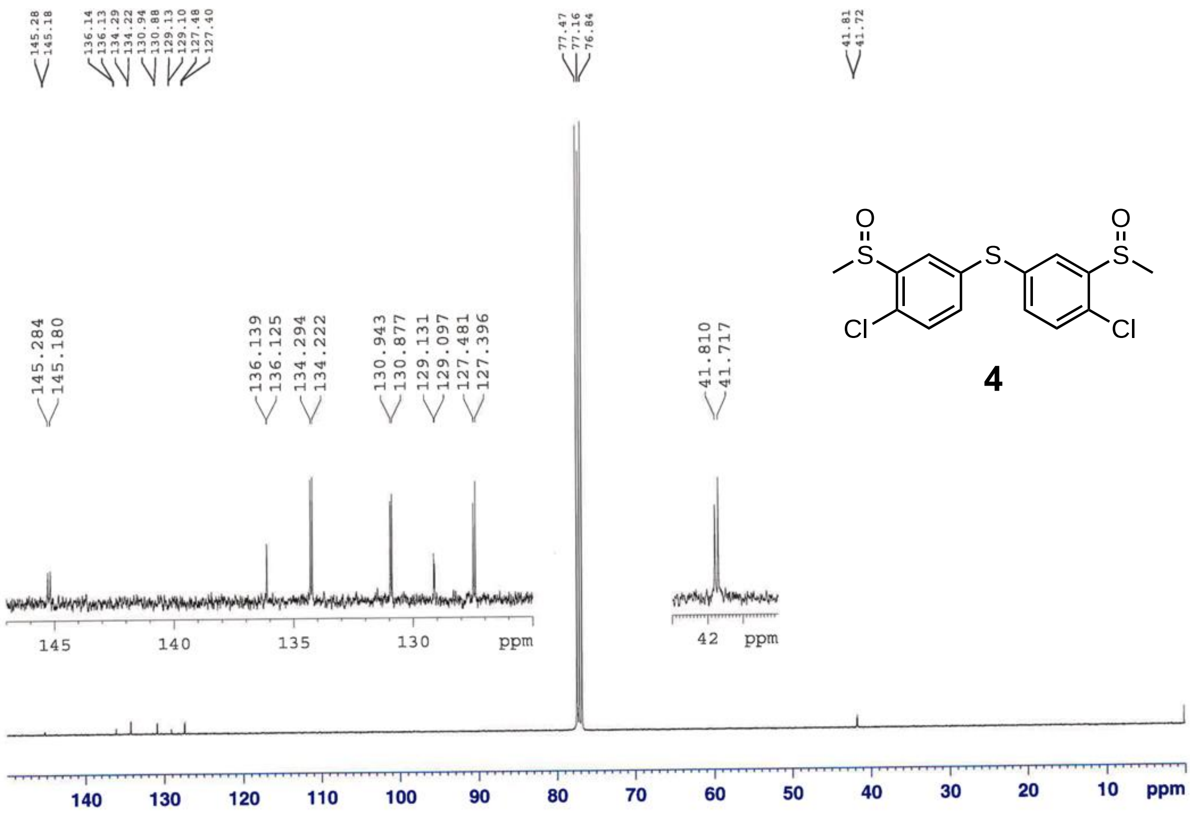

Figure S6. ^{13}C NMR spectrum of **4** (100 MHz, $CDCl_3$).

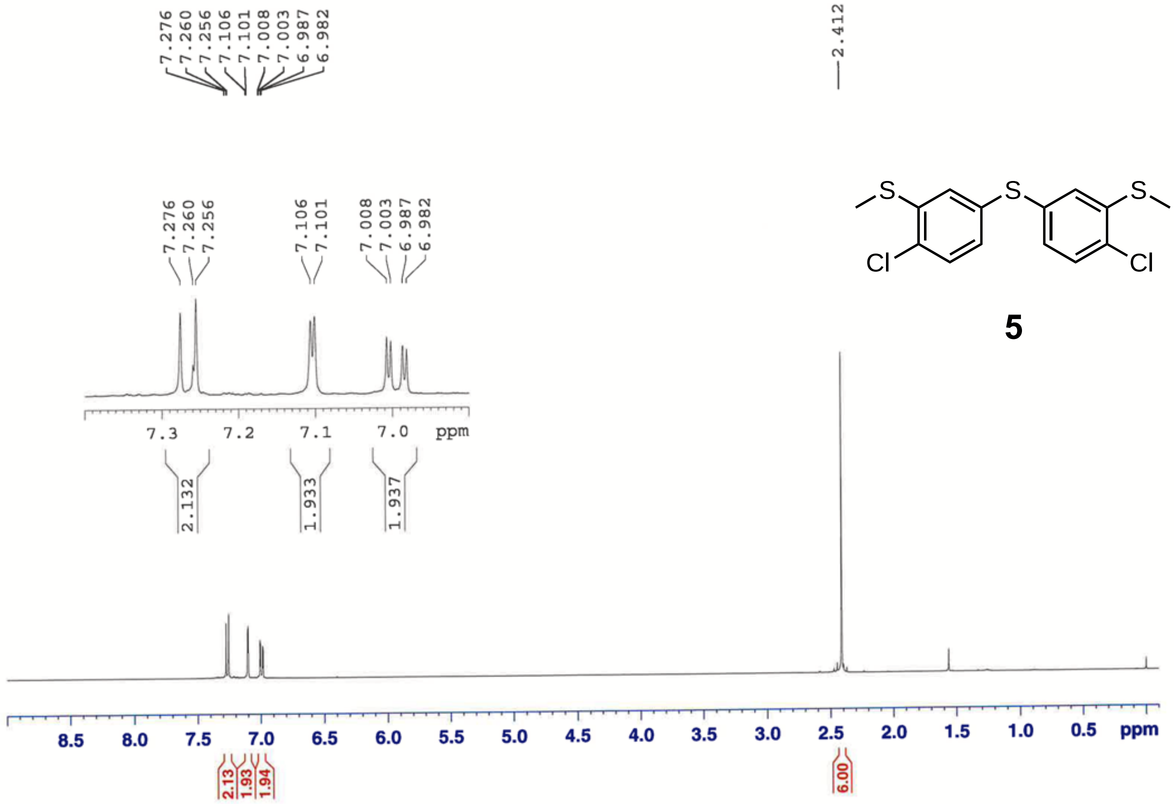

Figure S7. ^{1}H NMR spectrum of **5** (400 MHz, $CDCl_3$).

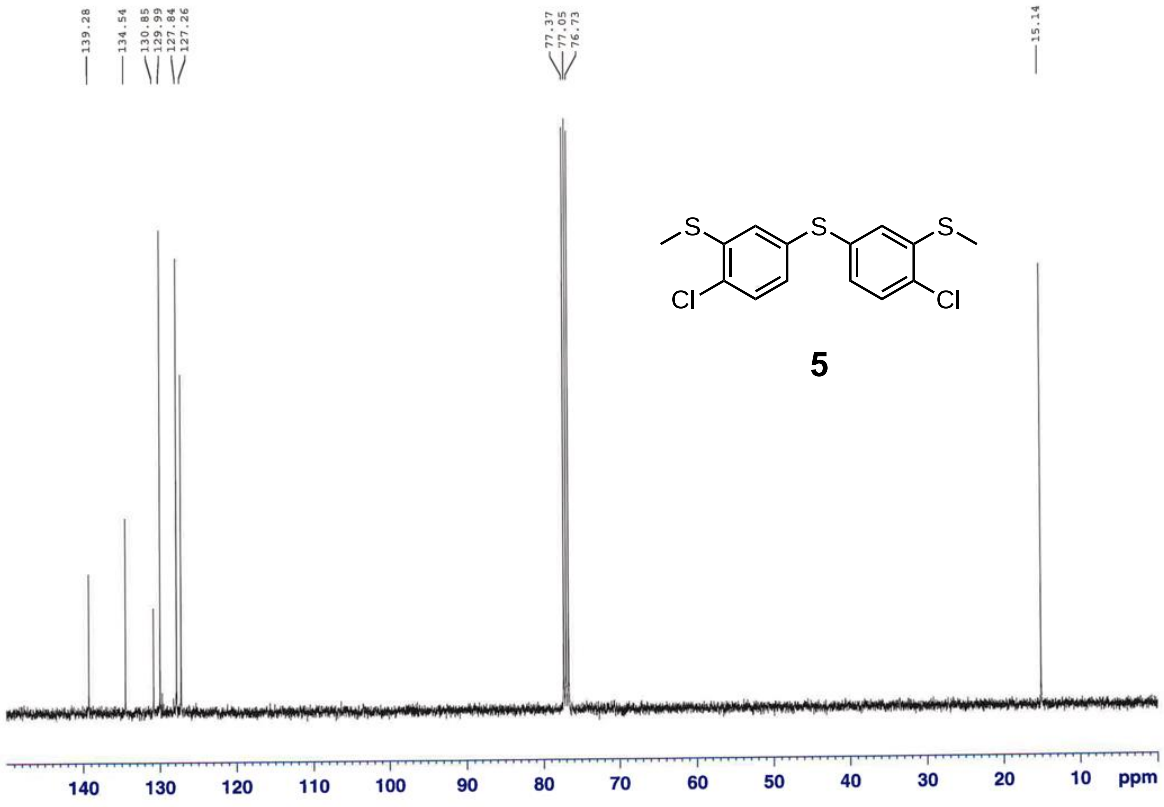

Figure S8. ^{13}C NMR spectrum of **5** (100 MHz, $CDCl_3$).

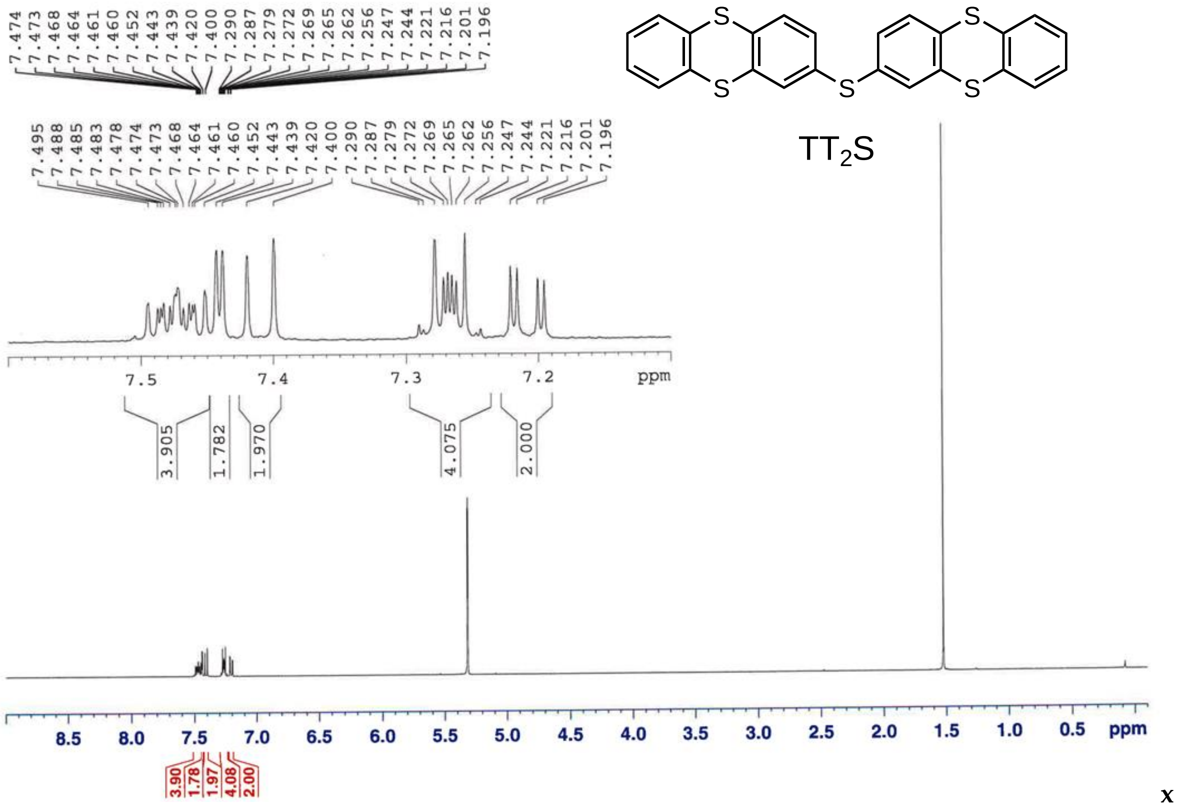

Figure S9. ^{1}H NMR spectrum of TT_2S (400 MHz, $CDCl_3$).

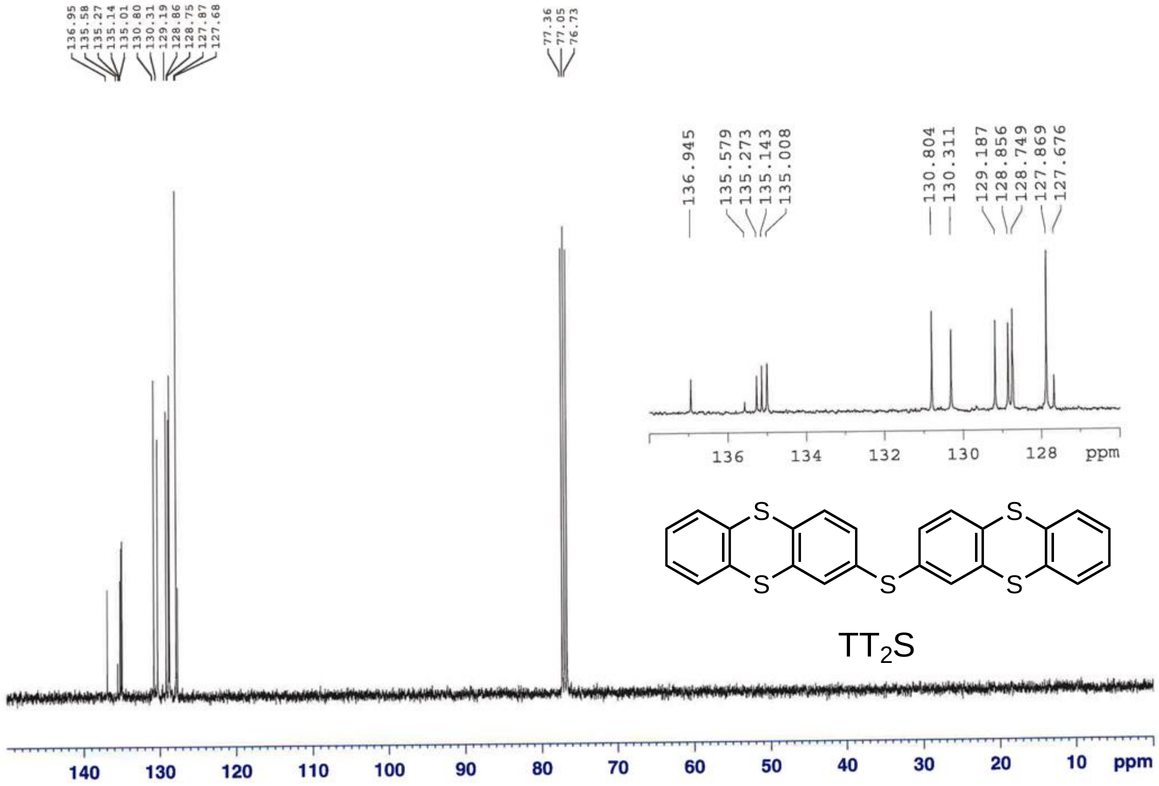

Figure S10. ^{13}C NMR spectrum of TT_2S (100 MHz, $CDCl_3$).

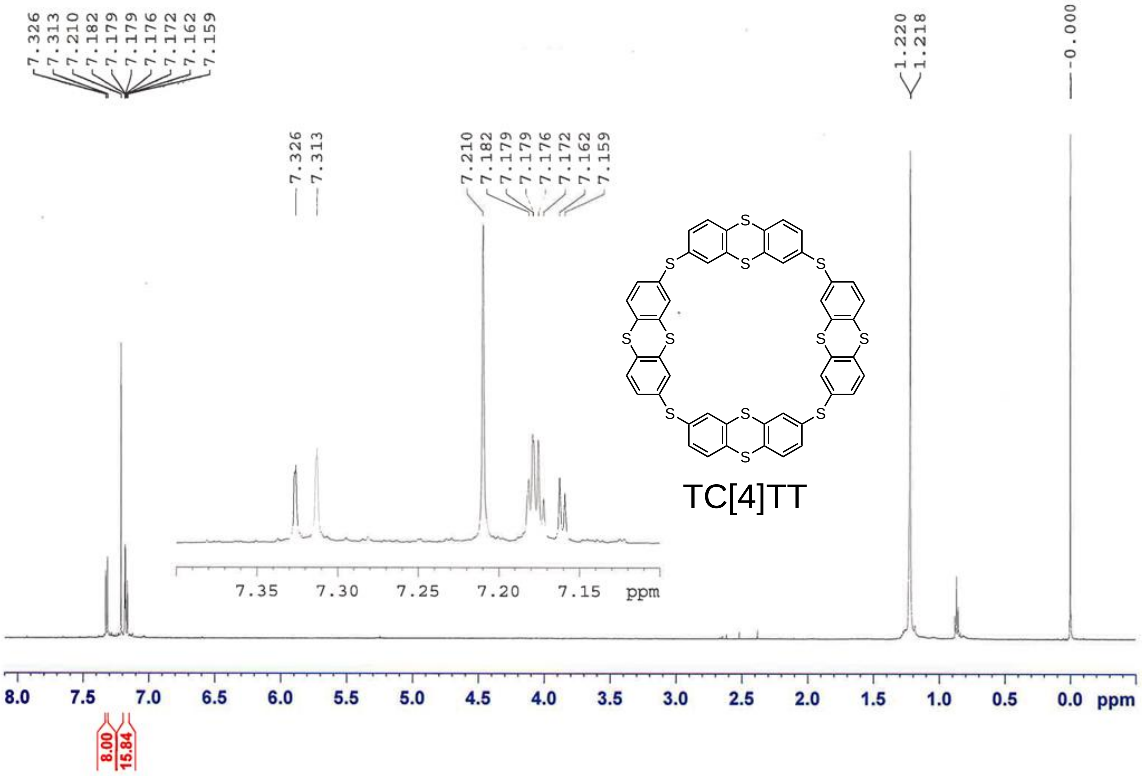

Figure S11. ^{1}H NMR spectrum of TC[4]TT (400 MHz, $CS_2/CDCl_3$).

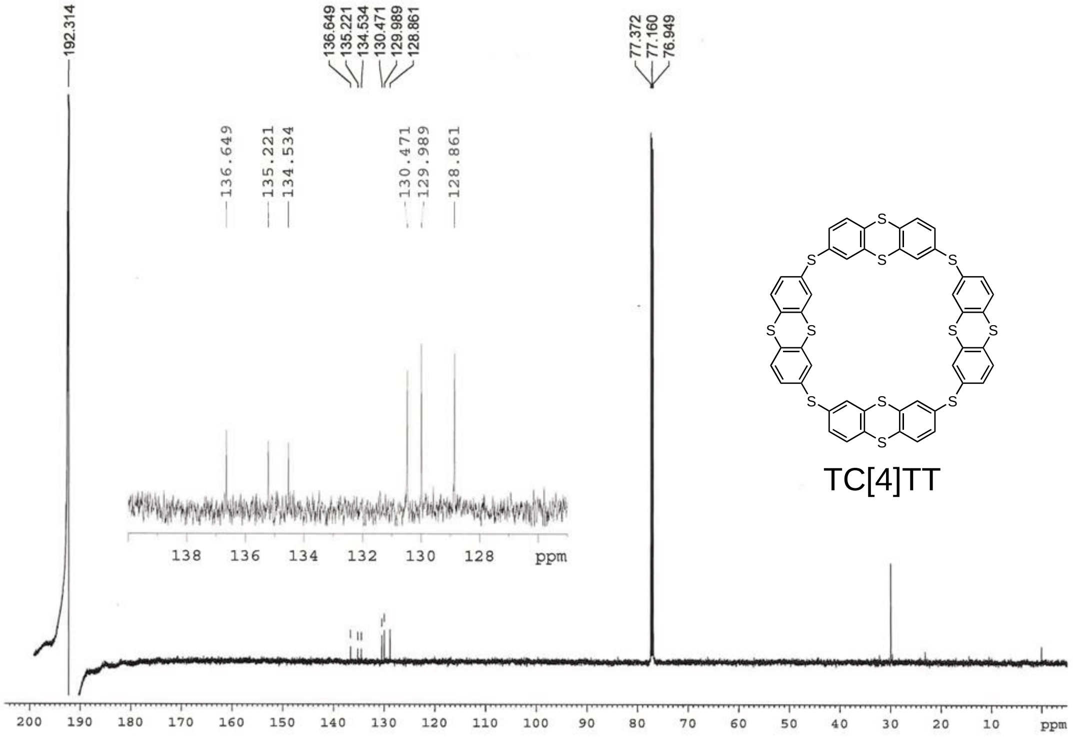

Figure S12. ^{13}C NMR spectrum of TC[4]TT (100 MHz, $CS_2/CDCl_3$).

2. ESIMS Spectra

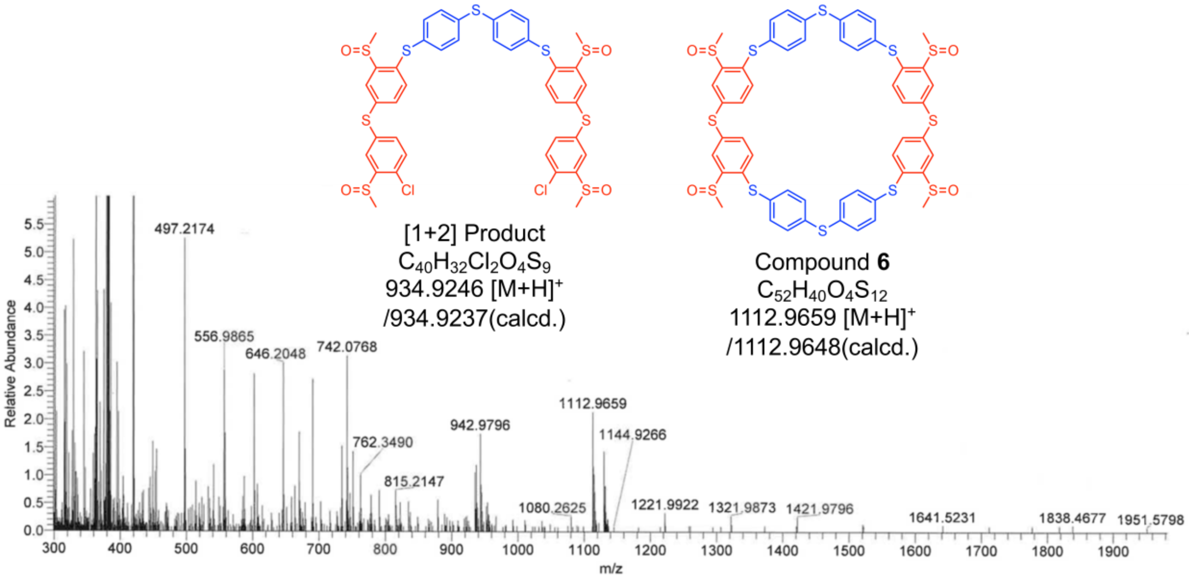

Figure S13. ESIMS spectra of crude products in macrocyclization of **4** and 4,4′-thiobisbenzenethiol

3. Crystal Data for TC[4]TT and TT_2S

Table S1. Crystal data for (TC[4]TT)$(CHCl_3)_2$, (TC[4]TT)$(CS_2)_2$, and TT_2S.

Compound	(TC[4]TT)$(CHCl_3)_2$	(TC[4]TT)$(CS_2)_2$	TT_2S
Formula	$C_{50}H_{26}Cl_6S_{12}$	$C_{50}H_{24}S_{16}$	$C_{24}H_{14}S_5$
T [K]	100.15	100.15	120.15
Crystal System	monoclinic	monoclinic	triclinic
Space Group	$I2$	$I2$	P-1
a [Å]	15.9127(4)	14.8376(5)	7.2043(3)
b [Å]	6.9189(2)	7.1679(2)	10.4023(3)
c [Å]	23.2775(6)	23.9132(8)	13.7311(3)
α [deg.]	90	90	82.435(2)
β [deg.]	94.443(2)	95.103(3)	89.282(3)
γ [deg.]	90	90	84.106(3)
V [Å^3]	2555.11(12)	2533.19(14)	1014.68(6)
Z	2	2	2
D_{calc} [gcm^{-3}]	1.591	1.491	1.514
μ [mm^{-1}]	7.952	6.634	5.325
F(000)	1240.0	1160.0	476.0
Crystal size [mm^3]	0.449×0.301×0.177	0.293×0.186×0.075	0.095×0.075×0.052
Reflection collected	6268	5981	8599
Independent reflection	3380	3317	3794
Parameters	307	298	262
Goodness-of-fit on F^2	1.067	1.091	1.030
$R1$, $wR2$ [I>2σ(I)]	0.0553, 0.1413	0.0472, 0.1207	0.0385, 0.0991
$R1$, $wR2$ [all data]	0.0560, 0.1422	0.0479, 0.1225	0.0414, 0.1010
ccdc number	2270345	2270346	2270347

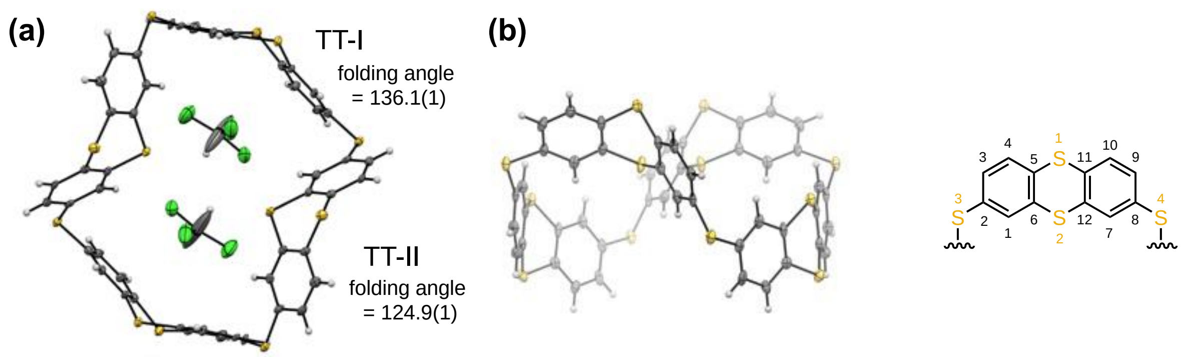

Figure S14. ORTEP illustration of (TC[4]TT)($CHCl_3$)$_2$: (a) top view and (b) side view. Ellipsoids are shown in a 50% probability.

Table S2. Selected bond lengths [Å] of TT units of TC[4]TT in (TC[4]TT)($CHCl_3$)$_2$.

Bond	TT-I	TT-II
C1–C2	1.415(8)	1.406(8)
C2–C3	1.380(11)	1.398(10)
C3–C4	1.393(10)	1.373(11)
C4–C5	1.408(9)	1.409(9)
C5–C6	1.378(11)	1.383(10)
C6–C1	1.379(10)	1.396(10)
C7–C8	1.392(10)	1.392(9)
C8–C9	1.378(12)	1.410(10)
C9–C10	1.404(9)	1.378(10)
C10–C11	1.392(10)	1.408(8)
C11–C12	1.394(11)	1.393(10)
C12–C7	1.411(8)	1.392(9)
C5–S1	1.772(7)	1.772(8)
C6–S2	1.783(6)	1.784(6)
C2–S3	1.774(7)	1.769(8)
C11–S1	1.776(6)	1.767(7)
C12–S2	1.760(7)	1.779(6)
C8–S4	1.772(6)	1.765(7)

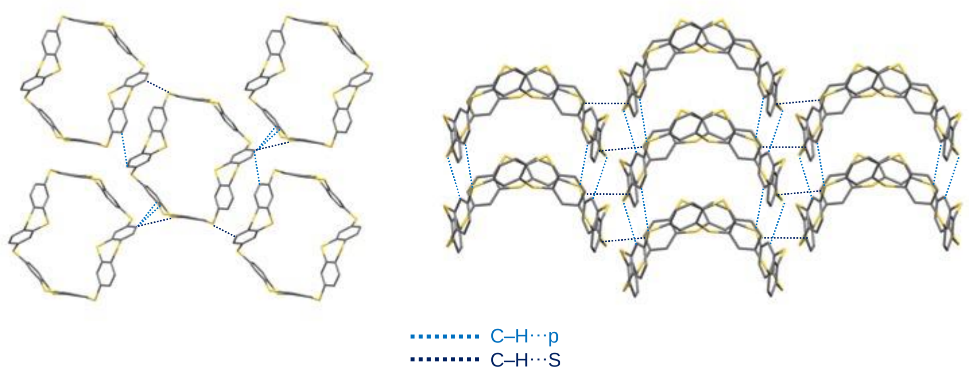

Figure S15. Packing diagrams of (TC[4]TT)($CHCl_3$)$_2$. $CHCl_3$ and H atoms are omitted for clarity.

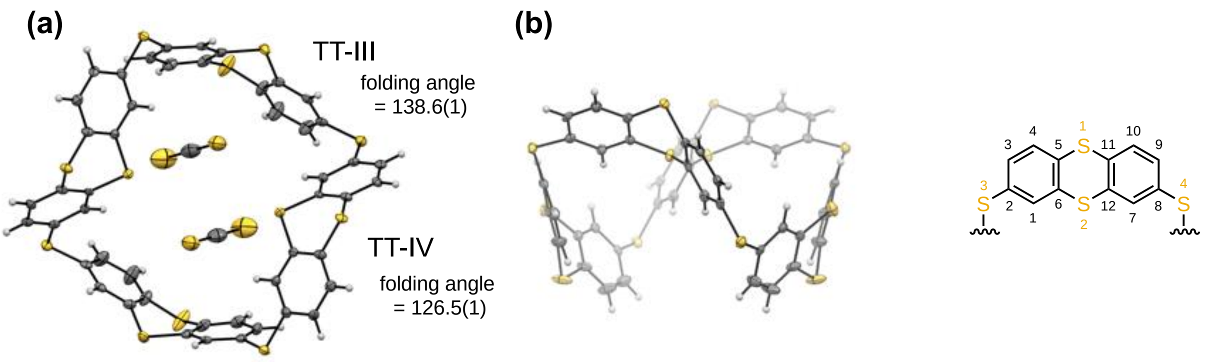

Figure S16. ORTEP illustration of $(TC[4]TT)(CS_2)_2$: (a) top view and (b) side view. Ellipsoids are shown in a 50% probability.

Table S3. Selected bond lengths [Å] of TT units of TC[4]TT in $(TC[4]TT)(CS_2)_2$.

Bond	TT-III	TT-IV
C1–C2	1.400(8)	1.405(8)
C2–C3	1.381(9)	1.394(8)
C3–C4	1.389(9)	1.380(9)
C4–C5	1.389(9)	1.404(7)
C5–C6	1.371(9)	1.403(8)
C6–C1	1.412(8)	1.393(9)
C7–C8	1.395(9)	1.391(8)
C8–C9	1.386(9)	1.396(8)
C9–C10	1.396(9)	1.383(8)
C10–C11	1.386(10)	1.394(8)
C11–C12	1.403(9)	1.390(8)
C12–C7	1.381(8)	1.387(7)
C5–S1	1.768(6)	1.775(6)
C6–S2	1.760(6)	1.763(5)
C2–S3	1.768(6)	1.775(6)
C11–S1	1.757(6)	1.776(6)
C12–S2	1.757(6)	1.781(6)
C8–S4	1.770(6)	1.775(6)

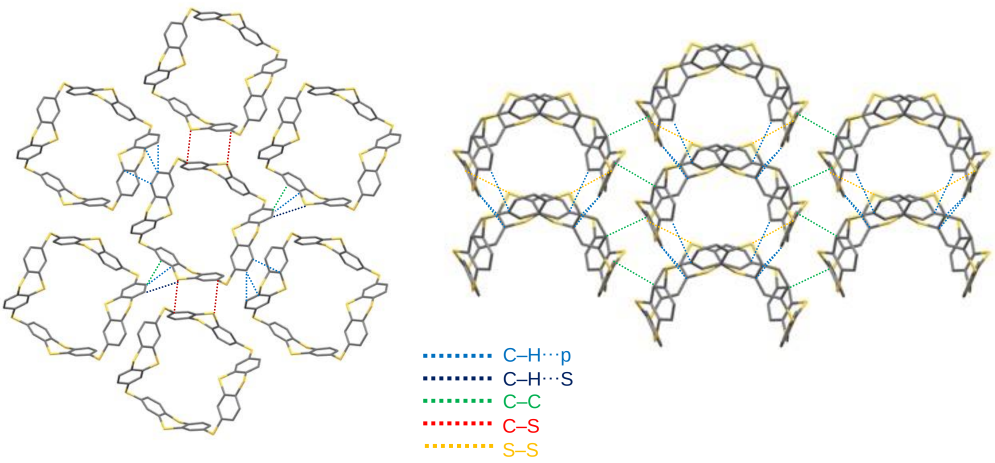

Figure S17. Packing diagrams of (TC[4]TT)(CS_2)$_2$. CS_2 and H atoms are omitted for clarity.

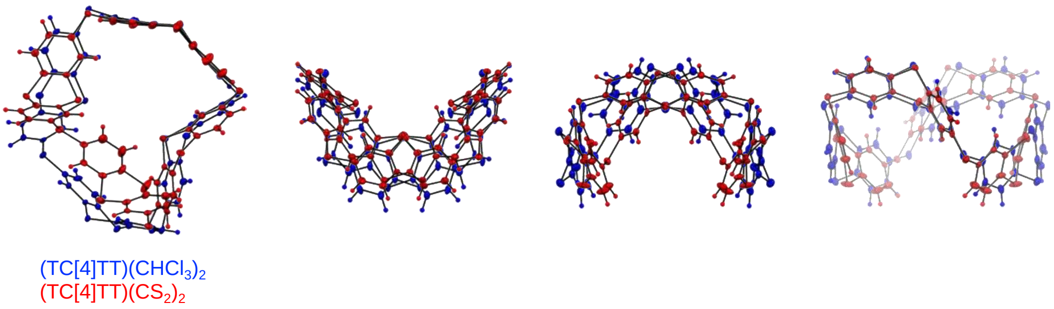

Figure S18. Structural difference of (TC[4]TT)($CHCl_3$)$_2$ and (TC[4]TT)(CS_2)$_2$.

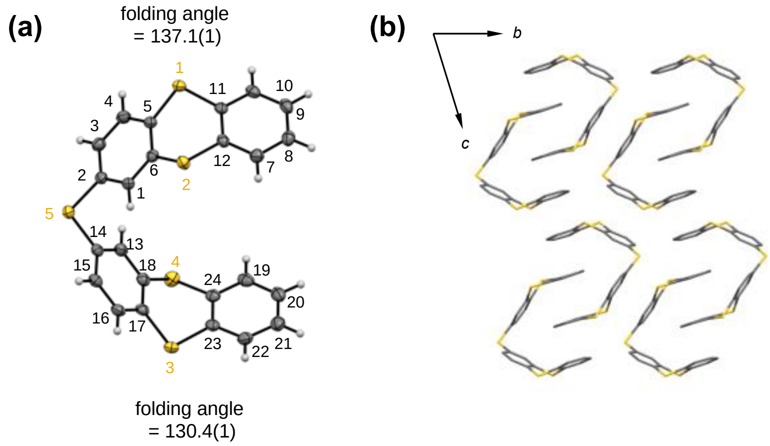

Figure S19. (a) ORTEP illustration of TT_2S. Ellipsoids are shown in a 50% probability. (b) Packing diagram of TT_2S. H atoms are omitted for clarity. Selected bond lengths [Å]: C1–C2 = 1.393(3); C2–C3 = 1.394(3); C3–C4 = 1.385(3); C4–C5 = 1.390(3); C5–C6 = 1.400(3); C6–C1 = 1.392(3); C7–C8 = 1.385(4); C8–C9 = 1.393(4); C9–C10 = 1.382(4); C10–C11 = 1.395(3); C11–C12 = 1.395(3); C12-C7 = 1.392(3); C5–S1 = 1.761(2); C6–S2 = 1.770(2); C2–S5 = 1.761(2); C13–C14 = 1.388(3); C14–C15 = 1.395(3); C15–C16 = 1.391(3); C16-C17 = 1.388(3); C17–C18 = 1.397(3); C18–C13 = 1.389(3); C19–C20 = 1.383(4); C20–C21 = 1.392(4); C21–C22 = 1.378(4); C22–C23 = 1.395(3); C23–C24 = 1.390(3); C24–C19 = 1.392(4); C17–S3 = 1.773(2); C18–S4 = 1.766(2); C14–S5 = 1.782(2); C23–S3 = 1.773(2); C24-S4 = 1.772(2).

4. Photophysical Properties

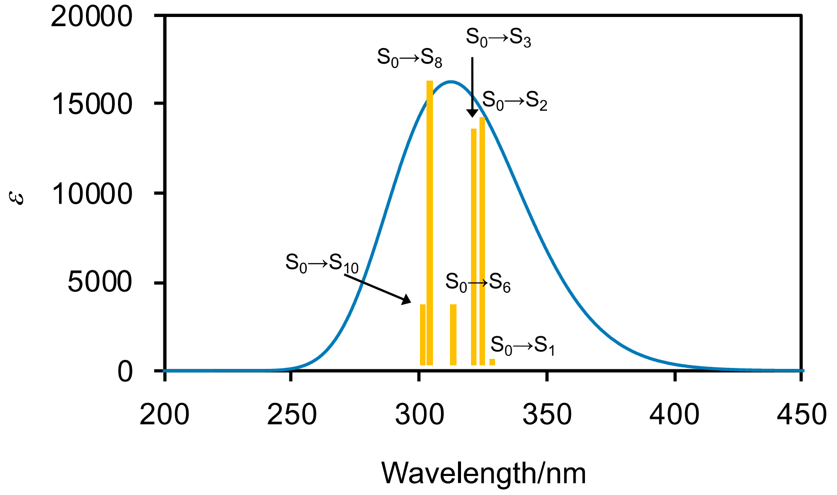

Figure S20. The simulated absorption spectrum of TC[4]TT by quantum chemical calculation (RB3LYP/6-31G (d,p) level).

Table S4. Calculated photophysical data for TC[4]TT at the RB3LYP/6-31G(d,p) level.

Electronic transition	Energy [eV]	Energy [nm]	Oscillator strengths	Major composition	CI coefficients
$S_0 \rightarrow S_1$	3.74	331	0.0005	HOMO−2 → LUMO+1 (22%)<br>HOMO → LUMO (52%)	0.33524<br>0.51070
$S_0 \rightarrow S_2$	3.79	327	0.0908	HOMO−2 → LUMO (38%)<br>HOMO → LUMO+1 (42%)	0.43852<br>0.45622
$S_0 \rightarrow S_3$	3.83	324	0.0880	HOMO−1 → LUMO (41%)<br>HOMO → LUMO+2 (29%)	0.45144<br>0.38295
$S_0 \rightarrow S_4$	3.87	321	0.0056	HOMO−3 → LUMO (23%)<br>HOMO−1 → LUMO+1 (16%)<br>HOMO−1 → LUMO+2 (19%)<br>HOMO → LUMO+3 (15%)	0.33686<br>−0.28489<br>−0.30472<br>−0.27166
$S_0 \rightarrow S_5$	3.92	316	0.0178	HOMO−1 → LUMO+1 (36%)<br>HOMO → LUMO+3 (19%)	0.42341<br>−0.30609
$S_0 \rightarrow S_6$	3.94	315	0.0295	HOMO−3 → LUMO+1 (15%)<br>HOMO−1 → LUMO (18%)	0.26969<br>−0.29695
$S_0 \rightarrow S_7$	4.06	305	0.0105	HOMO−3 → LUMO+3 (10%)<br>HOMO−2 → LUMO+2 (18%)<br>HOMO → LUMO+3 (11%)<br>HOMO → LUMO+7 (16%)	−0.22902<br>0.29696<br>−0.23421<br>−0.28001
$S_0 \rightarrow S_8$	4.07	305	0.1085	HOMO−3 → LUMO+2 (11%)<br>HOMO−2 → LUMO+3 (16%)<br>HOMO−1 → LUMO+7 (11%)<br>HOMO → LUMO+4 (11%)	−0.23061<br>0.28191<br>−0.23262<br>0.23788
$S_0 \rightarrow S_9$	4.10	302	0.0196	HOMO−3 → LUMO+3 (13%)<br>HOMO−2 → LUMO+1 (16%)<br>HOMO−2 → LUMO+2 (18%)	−0.25063<br>0.27950<br>0.29791
$S_0 \rightarrow S_{10}$	4.11	302	0.0299	HOMO−3 → LUMO+2 (15%)<br>HOMO−2 → LUMO+3 (12%)<br>HOMO−1 → LUMO+7 (15%)<br>HOMO → LUMO+6 (14%)	0.27557<br>−0.24388<br>−0.27316<br>0.26907

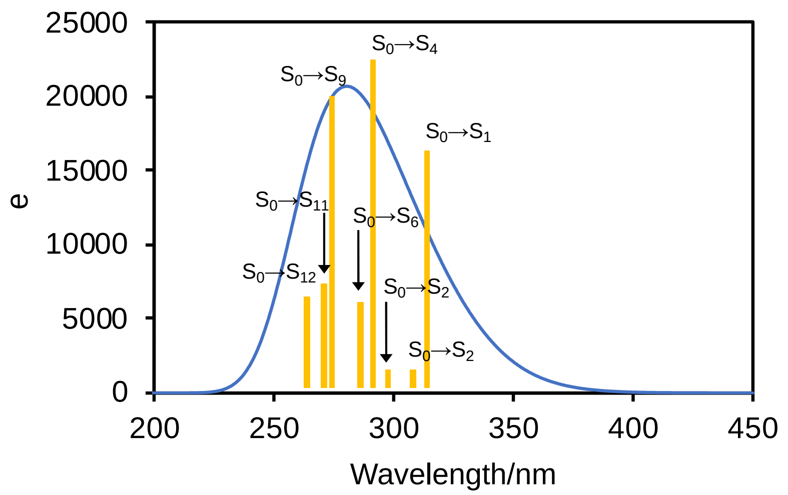

Figure S21. The simulated absorption spectrum of TT2S by quantum chemical calculation (RB3LYP/6-31G (d,p) level).

Table S5. Calculated photophysical data for TT2S at the RB3LYP/6-31G(d,p) level.

Electronic transition	Energy [eV]	Energy [nm]	Oscillator strengths	Major composition	CI coefficients
$S_0 \rightarrow S_1$	3.95	314	0.1195	HOMO−1 → LUMO+1 (10%)<br>HOMO → LUMO (80%)	0.22299<br>0.63296
$S_0 \rightarrow S_2$	4.02	309	0.0020	HOMO−1 → LUMO (63%)<br>HOMO → LUMO+1 (20%)	0.56346<br>0.31935
$S_0 \rightarrow S_3$	4.15	299	0.0032	HOMO−2 → LUMO (14%)<br>HOMO → LUMO+2 (66%)	0.26868<br>0.57607
$S_0 \rightarrow S_4$	4.26	291	0.1667	HOMO−2 → LUMO (36%)<br>HOMO−1 → LUMO+1 (27%)<br>HOMO → LUMO+2 (18%)	0.42493<br>−0.36652<br>−0.30135
$S_0 \rightarrow S_5$	4.26	291	0.0010	HOMO−1 → LUMO+2 (36%)<br>HOMO → LUMO+3 (37%)	0.42443<br>0.43165
$S_0 \rightarrow S_6$	4.31	288	0.0427	HOMO−1 → LUMO+1 (16%)<br>HOMO → LUMO+1 (64%)	−0.28490<br>0.56762
$S_0 \rightarrow S_7$	4.47	277	0.0013	HOMO−1 → LUMO+2 (25%)<br>HOMO → LUMO+3 (17%)<br>HOMO → LUMO+5 (21%)	0.35345<br>−0.29053<br>−0.32786
$S_0 \rightarrow S_8$	4.50	275	0.0276	HOMO−1 → LUMO+5 (17%)<br>HOMO → LUMO+6 (21%)	−0.28760<br>0.32628
$S_0 \rightarrow S_9$	4.52	274	0.1481	HOMO−2 → LUMO (29%)<br>HOMO−1 → LUMO+1 (35%)	0.37941<br>0.41870
$S_0 \rightarrow S_{10}$	4.53	273	0.0104	HOMO−1 → LUMO+6 (19%)<br>HOMO → LUMO+3 (22%)<br>HOMO → LUMO+5 (18%)	0.30809<br>0.32827<br>−0.30375
$S_0 \rightarrow S_{11}$	4.56	272	0.0585	HOMO−1 → LUMO+3 (39%)<br>HOMO−2 → LUMO+2 (16%)	0.44096<br>−0.28381
$S_0 \rightarrow S_{12}$	4.66	266	0.0547	HOMO−2 → LUMO+1 (62%)<br>HOMO−1 → LUMO+2 (15%)	0.55813<br>−0.27274

5. Theoretical Study

All calculations were performed with the Gaussian16 software package (Revision C.01) at the Research Center for Computational Science (Okazaki, Japan). Molecular geometries in the ground state (S_0) were optimized from the corresponding crystal structure as the initial structure using spin-restricted density functional theory (DFT) with the B3LYP functional and 6-31G(d,p) basis set. Vibration frequency calculations were performed to confirm that all optimized structures correspond to real local minima without any imaginary frequency. The stationary points of TC[4]TT, its structural isomer, and TT_2S were optimized with C_2 symmetry assumptions; TT was optimized with C_{2v} symmetry assumptions. The optimized Structures and molecular orbitals were illustrated using images displayed from Avogadro (ver. 1.2.0) software package. The absorption properties (Figure S20 and S21, Table S4 and S5) were obtained using time-dependent (TD) B3LYP/6-31G(d,p) at the ground state geometries. The energy levels of excited singlet (S_1) and triplet (T_n) states were estimated using TD-DFT at the B3LYP/6-31G(d,p) level. These calculation methods based on the standard B3LYP/6-31G(d,p) level of theory provide a suitable description for molecular geometries and excited states (S_n and T_n) of medium-sized and macrocyclic molecules. [S1-S3]

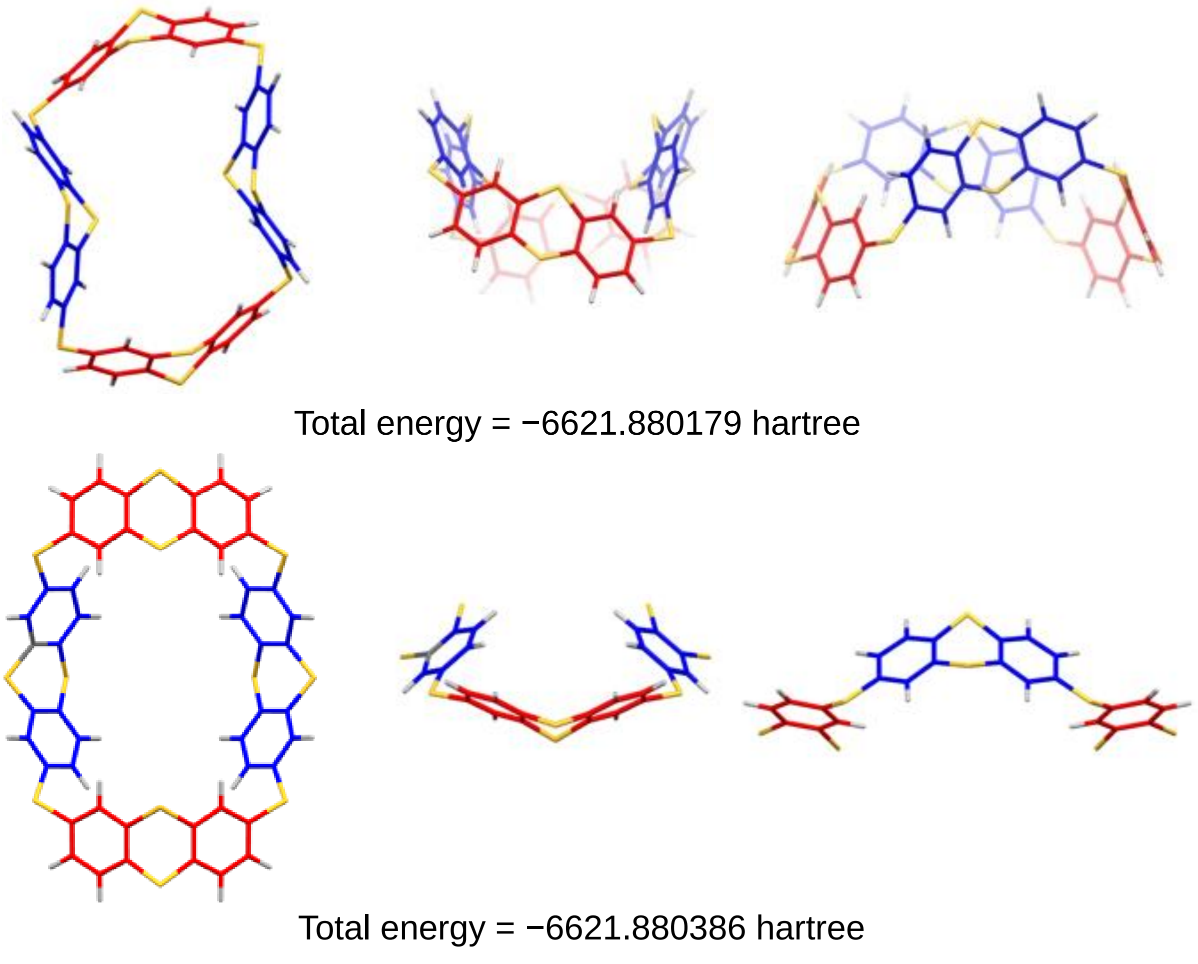

Figure S22. Optimized structures of TC[4]TT and its structural isomer at B3LYP/6-31G(d,p) level.

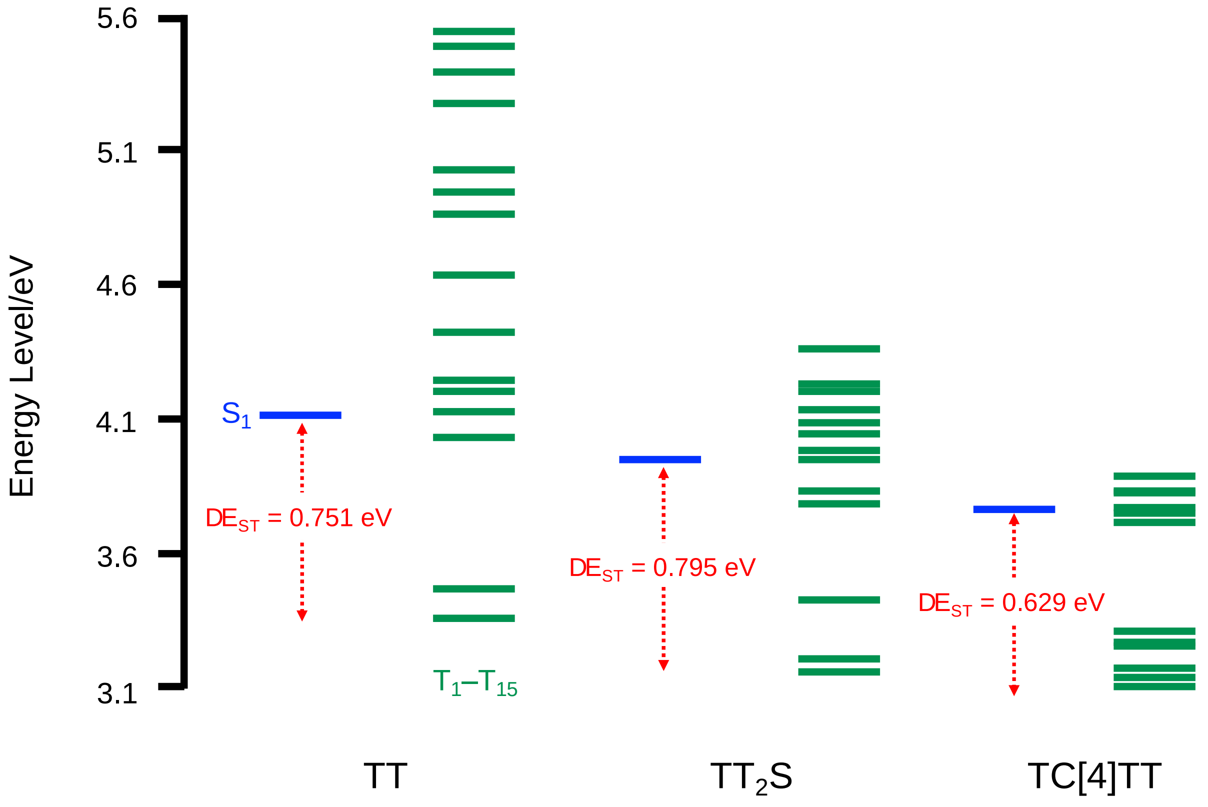

Figure S23. TD-DFT calculated energy diagrams at singlet (S_1) and triplets (T_n) for TC[4]TT, TT_2S, and TT at the B3LYP/6-31G(d,p) level.

Table S6. TD-DFT calculated energy levels [eV] at S_1 and T_n for TC[4]TT, TT_2S, and TT.

Excited State	TC[4]TT [eV]	TT_2S [eV]	TT [eV]
S_1	3.7449	3.9483	4.1067
T_1	3.1157	3.1533	3.3562
T_2	3.1158	3.2136	3.4741
T_3	3.1693	3.4208	4.0351
T_4	3.1698	3.4251	4.1246
T_5	3.2499	3.7791	4.1910
T_6	3.2532	3.8389	4.2394
T_7	3.2902	3.9380	4.4228
T_8	3.2932	3.9739	4.6329
T_9	3.7175	4.0520	4.8679
T_{10}	3.7176	4.0763	4.9398
T_{11}	3.7416	4.1464	5.0334
T_{12}	3.7423	4.1915	5.2762
T_{13}	3.8094	4.2100	5.3883
T_{14}	3.8149	4.2158	5.4828
T_{15}	3.8626	4.3568	5.5422

6. References

S1. T. Chen, L. Zheng, J. Yuan, Z. An, R. Chen, Y. Tao, H. Li, X. Xie, W. Huang. Understanding the Control of Singlet-Triplet Splitting for Organic Exciton Manipulating: A Combined Theoretical and Experimental Approach. *Sci. Rep.* **2015**, *5*, 10923.

S2. B. Milián-Medina, J. Gierschner, Computational design of low singlet-triplet gap all-organic molecules for OLED application. *Org. Electron.* **2012**, *13*, 985–991.

S3. H. Zhu, I. Badía-Domínguez, B. Shi, Q. Li, P. Wei, H, Xing, M. C. R. Delgado, F. Huang. Cyclization-Promoted Ultralong Low-Temperature Phosphorescence via Boosting Intersystem Crossing. *J. Am. Chem. Soc.* **2021**, *143*, 2164–2169.
